# A Comprehensive Review of the Correlations of Measurement Parameters among Modern Technologies for Sarcopenia Assessment

**DOI:** 10.14336/AD.2025.0145

**Published:** 2026-05-08

**Authors:** Dawei Zhang, Sai Kit Lam, Yongping Zheng

**Affiliations:** ^1^Department of Biomedical Engineering, The Hong Kong Polytechnic University, HKSAR, China; ^2^Research Institute for Smart Ageing, The Hong Kong Polytechnic University, HKSAR, China

**Keywords:** Bioelectrical impedance analysis, correlations, ultrasound, impaired mobility in older adults, muscle degradation, sarcopenia

## Abstract

Sarcopenia is characterized by a degeneration of muscle mass and strength, which impairs mobility and causes fragility in older adults. Modern assessment technologies include magnetic resonance imaging (MRI), computed tomography (CT), bioelectrical impedance analysis (BIA), dual-energy X-ray absorptiometry (DXA), and ultrasound (US). Despite the tremendous efforts made in the past to investigate the correlations among measurement parameters of these technologies, inconsistencies in the reported correlations persist, and there is no existing review article considering all these technologies for sarcopenia assessment, resulting in a lack of a common understanding about the correlations of these techniques. Herein, we conduct a comprehensive review to scrutinize the reported correlations between each pair of these five technologies from 51 publications in the literature. We classified these five technologies into two categories: image-based methods (IBMs), including CT, MRI, and US, and non-image-based methods (NIBMs), including DXA and BIA, resulting in ten pairs of correlations analyzed. The averaged correlations for NIBM-NIBM (BIA–DXA: 0.909) and IBM–IBM (MRI–CT: 0.978; MRI–US: 0.861; CT–US: 0.875; overall: 0.905) were high, while those for NIBM–IBM exhibited lower correlations with wider variations (DXA–CT: 0.834; BIA–CT: 0.824; BIA–MRI: 0.715; DXA–MRI: 0.834; DXA–US: 0.497; and BIA–US: 0.463; overall: 0.695). Generally, the correlations within the same category were greater than those between different categories. The dissimilar measurement locations and components could apparently cause lower correlations (e.g., BIA–US and DXA–US); the lower averaged correlations do not necessarily represent their low clinical value for sarcopenia assessments. By contrast, integrating NIBM and IBM may leverage the strengths of each technology, complementing their metrics, and bring a more holistic connection to sarcopenia. We hope to facilitate an enhanced understanding of the reported correlations from the literature, offering the community insightful recommendations for selecting technologies toward further research.

## 1. Introduction

Sarcopenia originates from the Greek roots "sarx" and "penia," meaning "flesh" and "loss," respectively [1]. It is characterized by the aging-associated progressive degradation of muscle mass, strength, and function [2]. In Asia, a 5.5%-25.7% prevalence of sarcopenia was reported in an epidemiological study conducted by the Asian Working Group for Sarcopenia (AWGS) using 2014 criteria [3]. In Hong Kong, approximately 9.4% of older male adults are facing sarcopenia [4]. These figures are anticipated to upsurge in the coming years in view of the rapidly expanding aging population worldwide. In fact, sarcopenia has posed grievous impacts on a broad array of adverse health outcomes, quality of life, and well-being of older adults [5] and has been associated with a variety of long-term conditions in mid-life [5, 6]. Consequently, in 2016, it was even recognized as having an independent health status with an International Classification of Diseases (ICD) code (ICD-10-CM (M62.84)) [7].

Modern diagnostic techniques for muscle quantity assessments include dual-energy X-ray absorptiometry (DXA), bioelectrical impedance analysis (BIA), computed tomography (CT), magnetic resonance imaging (MRI), and ultrasound imaging (US) [6]. DXA has been considered the most effective approach given its capability in estimating lean mass. BIA leverages the body’s electrical conductivity to indirectly estimate muscle mass, without involving ionizing radiation [8]. CT and MRI provide high-resolution images for precise assessments of muscle mass and other body tissues, but are mostly used in research and seldom in routine sarcopenia assessment workups. Of note, since 2018, US has been proposed as a clinical bedside toolkit for the assessments of both muscle quantity (such as muscle mass) and quality (such as muscle contraction) based on US echogenicity [9, 10]; and in 2020, the SARCUS (SARCopenia through UltraSound) Working Group further proposed an updated protocol for US-based sarcopenia assessments [11].

Over the past years, scientists have made tremendous efforts to investigate the correlations of the measurement parameters among the abovementioned modern diagnostic techniques, for the sake of revealing the validity of these approaches and favourable alternative strategies for muscle assessments to address the foreseeable demands under the rapidly growing aging population. Nevertheless, sarcopenia could be evaluated by reference to different muscle and fat-related parameters, both in the whole body and in specific areas. This leads to a huge heterogeneity in how studies measure and report their effects, including where the measurements were taken and whether they focused on muscle or fat. Such discrepancies between studies have erected a prime barrier to the community for harvesting a real understanding of the correlations among these diagnostic techniques, which may, in turn, have impeded the advancement or revolution of emerging desirable strategies for sarcopenia assessments in the future.

Therefore, it is imperative to conduct a comprehensive review of the correlations among all these five modern diagnostic technologies, yet a review is currently absent from the literature. In this review, we scrutinized the reported correlations by classifying the five techniques into two main categories: image-based methods (IBMs) and non-image-based methods (NIBMs), and by sorting all the measurement parameters in accordance with their measurement locations and components. Our overarching goals are to shed more light on the overall pattern of correlations among the five diagnostic techniques, offer the community insights for the choice of technologies toward future research, and ameliorate the sarcopenia assessments in the long run.

The overall structure of this article is summarized as follows: Section 2 provides a brief introduction to the current diagnostic criteria and methods for sarcopenia measurements. Section 3 scrutinizes the correlations among the five diagnostic techniques under the categories of IBM–IBM, NIBM–NIBM, and NIBM–IBM correlations. Section 4 offers an in-depth discussion of insights and recommendations for future research.

## 2. Current Diagnostic Criteria and Methods for Sarcopenia

Globally, three major working groups, the AWGS [3], the European Working Group on Sarcopenia in Older People (EWGSOP2) [12], and the International Working Group on Sarcopenia (IWGS) [13], provide an evidence-based consensus and recommendations for the definition, diagnostic approaches, and criteria for sarcopenia; the corresponding criteria are summarized in Supplementary Tables 1-3. According to these international bodies, MRI, CT, US, BIA, and DXA are the five key modern technologies for sarcopenia assessments, while it is worth noting that BIA and DXA are the most typically recommended approaches. The pros and cons of each of these approaches are displayed in Supplementary Table 4.

In this review, we classified these five modern approaches into two categories: (i) image-based methods (IBMs) for MRI, CT, and US, (ii) non-image-based methods (NIBMs) for BIA and DXA. Notably, DXA is classified as an NIBM in this article because of the fact that DXA-derived body composition indexes are eventually computed using the absorption parameters instead of using morphological image parameters, despite involving the use of X-ray imaging techniques [9, 14].

## 3. Comparisons of the Correlations among Modern Technologies

A total of 51 related studies from the current body of literature were reviewed and analyzed regarding the detailed characteristics of the 51 included studies. We conducted the literature search in the PubMed and Google Scholar databases for studies published between 2007 and 2023. The search strategy included the following keywords in the title or abstract: "Sarcopenia" and/or "Muscle", combined with at least two of the five technologies: BIA (Bioelectrical Impedance Analysis), DXA (Dual-Energy X-ray Absorptiometry), MRI (Magnetic Resonance Imaging), CT (Computed Tomography), or US (Ultrasound) imaging. Both the abbreviations and full names of the technologies were used as search terms. For example, “BIA”, “DXA”, and “sarcopenia” were searched in the search query box. Our inclusion criteria were as follows: (1) studies involving patients aged 18 years or older; (2) studies reporting correlation coefficients (r) using the Pearson's, Spearman's, Kendall's, or ICC (Intraclass Correlation Coefficient) methods; (3) studies conducting clinical observational studies; and (4) studies published in English. The screening process involved initial screening with titles and abstracts of potentially relevant studies and full-text reviews to ensure that they met the inclusion criteria. From the initial search of the databases, we obtained 101 articles with no publication time limits. After removing duplicated articles, we screened 91 articles with titles and abstracts. We removed articles with animal models and irrelevant technologies such as handgrips and obtained 77 articles for full-text article assessment. We removed articles with clinical animal experiments conducted. In the end, we included 51 articles for the final analysis. The sample size, distribution of ages, types of modern technologies studied, subjects’ baseline health conditions, and types of correlation analysis for the studied pairs are provided in Supplementary Table 5. As per the categorization of IBMs and NIBMs defined in Section 2, there were a total of ten pairs of correlations for comparison: one pair for NIBM–NIBM comparison (BIA vs DXA); three pairs for IBM–IBM (MRI–CT, CT–US and MRI–US); and six pairs for NIBM–IBM (BIA–MRI, BIA–CT, BIA–US, DXA–MRI, DXA–CT, and DXA–US). The abbreviations of the related terminologies are listed in Supplementary Table 6.

Given the fact that the measurement parameters derived from these five modern technologies were highly heterogeneous between the studies, particularly in terms of the body sites and components involved in the measurements, for the purpose of facilitating the readers of this review article to understand and interpret it, we classified all the measurement parameters based on two key aspects: measurement locations and measurement components. For the measurement locations, we denoted the parameters that were measured from a holistic body scale as “Global” (G), and those measured at specific body parts, for instance, a specific muscle or a specific slice of lumbar area, or a particular region of the abdomen or a leg, etc. were denoted as “Partial” (P). For the measurement components, we denoted the parameters that were measured in muscle tissue as “muscle-related” (M), and those measured in fatty tissue were denoted as “fat-related” (F). Though muscle-related parameters are important for sarcopenia assessment, fat-related parameters are also important since they are essential to assess sarcopenic obesity [3, 12, 15, 16]. AWGS and EWGSOP mentioned that obesity is crucial for elderly people who already face the condition of sarcopenia; thus, obesity is crucial to be taken into consideration when measuring sarcopenia. In light of this statement, we included fat-related parameters for consideration since incorporating only muscle-related parameters is insufficient for overall muscle assessment. For cases where the parameters were neither measured from pure fat nor muscle tissues, such as body mass index (BMI), lean mass (LM), fat-free mass (FFM), soft tissue, etc., we defined the parameters based on their measurement locations, either as “Global index” or “Partial index”. With the above definitions, we denoted arm lean mass, for instance, PG where the first letter P refers to the partial body location of the arm, and the second letter G stands for the global measurement component of lean mass. Similarly, we denoted fat mass as GF, rectus femoris muscle thickness as PM (partial-muscular), BMI as G, and similarly for all the other measurement parameters. A summary is given in Supplementary Table 7. According to the pros and cons of each approach, a recommendation table showing the usage of each approach under different scenarios is shown in Supplementary Table 8, where the approaches are recommended based on patient characteristics, available resources, and the specific assessment goals of testing.

In terms of the types of correlations among the included studies, we report Pearson’s correlation coefficient (PCC), the Spearman correlation coefficient (SCC), and the intra-correlation coefficient (ICC), based on the fact that these correlation coefficients are the most widely analyzed metrics for demonstrating the relationship and agreements between two diagnostic approaches. Notably, we selected the literature with significant correlation comparisons with a p-value < 0.05.

**Figure 1.**
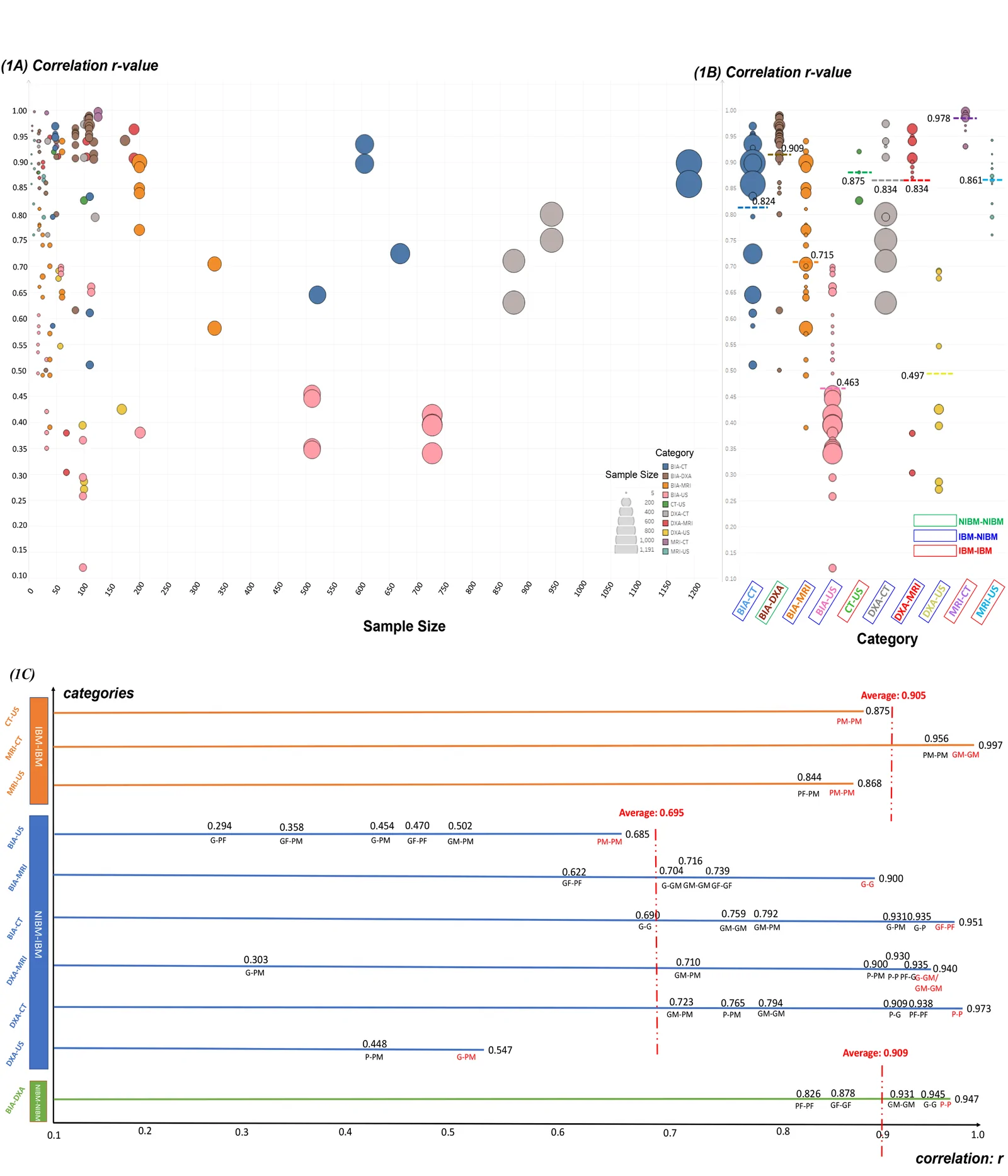
**A bubble plot providing an overview of the reported correlations among the ten pairs studied, with the x-axis indicating the sample size (A) and the ten pairs of diagnostic methods (B)**. In both graphs, each bubble represents a correlation assessment. As the studies are reporting more than one correlation value, the total number of bubbles is greater than the number of included studies. (C) A bar graph providing a close look at the correlation scores for specific measurement pairs, under IBM–IBM (Top: orange), NIBM–IBM (Middle: blue), and NIBM–NIBM (Bottom: green). The red lines show the average correlations in IBM–IBM, NIBM–IBM, and NIBM–NIBM. Abbreviations of the measurement pairs are provided at the top. G-G: Global-Global; P-P: Partial-Partial; G-P: Global-Partial; G-PM: Global-Partial Muscle; P-PM: Partial–Partial Muscle; G-GM: Global–Global Muscle; GM-GM: Global Muscle-Global Muscle; GF-GF: Global Fat-Global Fat; PM-PM: Partial Muscle-Partial Muscle; PF-PM: Partial Fat- Partial Muscle; GM-PM: Global Muscle- Partial Muscle; GF-PM: Global Fat- Partial Muscle; GF-PF: Global Fat- Partial Fat.

Notably, most of the reported correlations were derived from small cohorts of less than 200 subjects (Figure 1A). There exist varying degrees of variations in the reported correlation values between the studies for each pair of diagnostic methods (Fig. 1B), with their average values (SD) being 0.909 (SD=0.103) for BIA–DXA, 0.875 (SD=0.039) for CT–US, 0.978 (SD=0.022) for MRI–CT, 0.861 (SD=0.053) for MRI–US, 0.824 (SD=0.147) for BIA–CT, 0.715 (SD=0.147) for BIA–MRI, 0.463 (SD=0.143) for BIA–US, 0.834 (SD=0.105) for DXA–CT, 0.834 (SD=0.204) for DXA–MRI, and 0.497 (SD=0.166) for DXA–US (Fig. 1B), where MRI–CT achieved the highest average correlation and the BIA–US and DXA–US pairs presented the lowest corrections.

The Figure 1C provides a close look at the correlation scores for specific measurement pairs, under IBM–IBM, NIBM–IBM, and NIBM–NIBM, separately. Overall, it shows that IBM–IBM and NIBM–NIBM exhibit stronger correlations than NIBM–IBM. Of note, although IBM and NIBM are generally less correlated with each other, the NIBM–IBM embodies the greatest variability in the measurement pairs, in terms of measurement locations and components (Fig. 1C). As such, the correlations were analyzed between different body scales and body components, such as GF-PM in BIA–US where the correlations were analyzed between global fat parameters and partial muscle parameters, which may be attributed to the generally lower correlations than for the IBM–IBM and NIBM–NIBM. More elaborations are given in Sections 3.1-3.3, where the corresponding pairs of diagnostic methods are discussed. A detailed description of the sample size and gender distribution is included in a pie chart in Supplementary Figure 1.

### 3.1 NIBM vs NIBM (BIA–DXA)

Both DXA and BIA belong to the NIBM category. Among the 51 studies, there are six reported correlations of the derived parameters [14, 15, 17-20]. Both techniques have been deployed to examine global or partial composition indexes, such as body mass, Fat Mass (FM), Appendicular Lean Mass (ALM), Free Fat Mass (FFM), and Percentage Body Fat (BFP). Table 1 displays the reported correlations between BIA parameters and DXA parameters. The correlations of sarcopenia indexes within the NIBM category (i.e., BIA–DXA) are generally high to excellent (Avg: 0.909, Range: 0.500-0.989, SD: 0.103) (Fig. 1B, Table 1).

**Table 1:** The reported correlations of indexes derived between BIA and DXA.

Author	Year	BIA Parameter	DXA Parameter	Correlation (r)	Sample Size and Gender Distribution
**Yi et al.** [15]	2022	FFM (Inbody 970) (G: Global)	FFM(G: Global)	0.989	109 subjects (55 males, 54 females)
		BFP (Inbody 970) (GF: Global, Fat)	BFP(GF: Global, Fat)	0.964	
		ALM (Inbody 970) (G: Global)	ALM(G: Global)	0.971	
		FFM (Body Water Analysis, Clamp) (G: Global)	FFM(G: Global)	0.984	
		BFP (Body Water Analysis, Clamp)(GF: Global, Fat)	BFP(GF: Global, Fat)	0.947	
		ALM (Body Water Analysis, Clamp) (G: Global)	ALM(G: Global)	0.971	
		FFM (Body Water Analysis, Adhesive) (G: Global)	FFM(G: Global)	0.985	
		BFP (Body Water Analysis, Adhesive) (GF: Global, Fat)	BFP(GF: Global, Fat)	0.953	
		ALM (Body Water Analysis, Adhesive) (G: Global)	ALM(G: Global)	0.969	
**Cruz Rivera et al.** [17]	2022	FM(GF: Global, Fat)	FM(GF: Global, Fat)	0.940	50 subjects (48 males, 2 females)
		FFM(G: Global)	FFM(G: Global)	0.910	
		TPF(GF: Global, Fat)	TPF(GF: Global, Fat)	0.800	
		FFM(G: Global)	FFM(G: Global)	Obese: 0.920	
		FFM(G: Global)	FFM(G: Global)	Non-obese: 0.860	
		TPF(GF: Global, Fat)	TPF(GF: Global, Fat)	Obese: 0.850	
		TPF(GF: Global, Fat)	TPF(GF: Global, Fat)	Non-obese: 0.500	
		FM(GF: Global, Fat)	FM(GF: Global, Fat)	Obese: 0.960	
		FM(GF: Global, Fat)	FM(GF: Global, Fat)	Non-obese: 0.840	
**Achamrah,et al.** [14]	2018	FM(GF: Global, Fat)	FM(GF: Global, Fat)	0.955	3,660 subjects (653 males and 3,002 females)
		FFM(G: Global)	FFM(G: Global)	0.897	
**Buch et al** [18]	2022	Arms Lean Body Mass(P: Partial)	Arms Lean Body Mass(P: Partial)	0.933	84 subjects (35 males, 49 females)
		Legs Lean Body Mass(P: Partial)	Legs Lean Body Mass(P: Partial)	0.957	
		Trunk Lean Body Mass(P: Partial)	Trunk Lean Body Mass(P: Partial)	0.950	
		ASMI(GM: Global, Muscle)	ASMI(GM: Global, Muscle)	0.965	
		PF(GF: Global, Fat)	PF(GF: Global, Fat)	0.954	
		Arms Fat Mass(PF: Partial, Fat)	Arms Fat Mass(PF: Partial, Fat)	0.615	
		Legs Fat Mass(PF: Partial, Fat)	Legs Fat Mass(PF: Partial, Fat)	0.907	
		Trunk Fat Mass(PF: Partial, Fat)	Trunk Fat Mass(PF: Partial, Fat)	0.955	
Vermeiren et al. [19]	2019	ALM (G: Global)	ALM (G: Global)	0.942	174 subjects (91 males, 83 females)
**Bosaeus et al.** [20]	2013	SMM (Tengvall et al.*) (GM: Global, Muscle)	SMM (GM: Global, Muscle)	0.915	117 subjects (72 males, 45 females)
		SMM(Kyle et al.*) (GM: Global, Muscle)	SMM (GM: Global, Muscle)	0.939	
		SMM(Janssen et al.*) (GM: Global, Muscle)	SMM (GM: Global, Muscle)	0.906	

Abbreviations: FFM: Fat-Free Mass; BFP: Body Fat Percentage; ALM: Appendicular Lean Mass; FM: Fat Mass; TPF: Total Percentage Fat; ASMI: Appendicular Skeletal Mass Index; PF: Percentage Fat; SMM: Skeletal Muscle Mass; ALM: Appendicular Lean Mass

* Tengvall, Kyle, and Janssen are three authors who generated the equation for calculating skeletal muscle mass.

The majority of the parameters involved in this category were derived from global body compositions and related to fatty components (Table 1). Only three studies analyzed partial body compositions, yielding a correlation of 0.957 for legs lean body mass, 0.950 for trunk lean body mass, and 0.933 for arms lean body mass [18].

Under different BIA devices (with varying frequencies), the reported correlations between BIA and DXA appear to be stable for global body fatty compositions (BFP: 0.947-0.964) and global body muscular composition (ALM: 0.969-0.971) [15] (Table 1). High-frequency BIA (HF-BIA) generates higher correlations with DXA [15]. Second, with older adults suffering from chronic obstructive pulmonary disease (COPD) or type 2 diabetes, the BIA–DXA correlations are generally greater than 0.900 for GF, GM, and PF compositions [18]. Third, obese subjects generated stronger correlations compared to non-obese subjects[17]. For example, the BIA–DXA correlation of total percentage fat (TPF) in non-obese subjects was 0.500 but reached 0.850 for obese patients [17].

Overall, the BIA–DXA comparison tends to have a high correlation over 0.900. According to Figure 1C, the average correlation of PF-PF is 0.826 [18], 0.878 for GF-GF [14, 15, 17, 18], 0.931 for GM-GM [18, 20], 0.945 for G-G [14, 15, 17, 19], and 0.947 for P-P [18]. The high correlations could be partly attributed to the fact that the pairs of measurement parameters at least share similar measurement locations.

### 3.2 IBM vs IBM

MRI, CT, and US belong to the IBM category. Table 2A-2C displays the reported correlations for MRI–CT, MRI–US, and CT–US pairs of diagnostic methods from the literature. The correlations of the IBM category are generally high to excellent, with an average correlation of 0.978 (range: 0.930-0.997, SD: 0.022) for MRI–CT, 0.861 (range: 0.760-0.942. SD: 0.053) for MRI–US, and 0.875 (range: 0.826-0.920, SD: 0.039) for CT–US pairs (Fig. 1B). As illustrated in Table 2A-2C, IBMs provide measurement results from specific muscles or fat tissues, where segments are often drawn around the borders of the interested muscles or fat tissues. Thus, muscle thickness (MT) and cross-sectional area (CSA) are the most popular indicators for IBM–IBM correlation assessments.

**Table 2:** The reported correlations of indexes derived between MRI and CT.

Author	Year	MRI Parameter	CT Parameter	Correlation (r)	Sample Size and Gender Distribution
**Khan et al.** [21]	2019	Abdominal SMA(PM: Partial, Muscle)	Abdominal SMA(PM: Partial, Muscle)	0.997	19 subjects (15 males, 4 females)
**Zwart et al.** [22]	2020	Total CSA of head-and-neck muscles at the C3 level(PM: Partial, Muscle)	Total CSA of head-and-neck muscles at the C3 level(PM: Partial, Muscle)	0.987	125 subjects (90 males, 35 females)
		SMI(GM: Global, Muscle)	SMI(GM: Global, Muscle)	0.997	
**Faron et al.** [23]	2020	Paraspinal SMA(PM: Partial, Muscle)	Paraspinal SMA(PM: Partial, Muscle)	0.930	50 subjects (31 males, 19 females)
**Lee et al.** [24]	2021	MT of Temporalis(PM: Partial, Muscle)	MT of Temporalis(PM: Partial, Muscle)	0.894	106 subjects (84 males and 22 females)
**Dupont et al.** [25]	2001	MT of supraspinatus(PM: Partial, Muscle)	MT of supraspinatus(PM: Partial, Muscle)	0.960	6 subjects (3 males, 3 females)
		MT of deltoid muscle(PM: Partial, Muscle)	MT of deltoid(PM: Partial, Muscle)	0.970	
**Wang et al.** [26]	2021	Abdominal SMA(PM: Partial, Muscle)	Abdominal SMA(PM: Partial, Muscle)	0.995	32 subjects (15 males, 17 females)

Abbreviations: SMA: Skeletal Muscle Area; CSA: Cross-Sectional Area; SMI Skeletal Muscle Mass Index; MT: Muscle Thickness

MRI–CT: Among the 51 studies, six reported correlations of the derived parameters between these two diagnostic methods [21-26]. Both MRI and CT are considered the gold standard for assessing sarcopenia [8, 27]; they achieved the highest reported correlation among the ten studied pairs of diagnostic methods (Fig. 1B), yielding an average correlation of 0.978 (SD=0.022). MRI and CT focus on the measurement of muscular components. Among these parameters, it appears that the reported MRI–CT correlation was the highest for abdominal SMA, SMI, and the total CSA of muscle at the C3 level of the head-and-neck region (range: 0.987-0.997) [21, 22, 26], followed by supraspinatus/deltoid MT (range: 0.960-0.970) [25], paraspinal SMA (0.930) [23], and temporalis muscle thickness (TMT)(0.894) [24] (Table 2). Although the sample size of these studies varied from six to 125 subjects, the reported correlations appear to be stably high regarding whether the subjects were beyond healthy or diseased populations (Table 2, Supplementary Table 7). According to Figure 1C, the average correlation of PM-PM is 0.956 [21-26], and 0.997 for GM-GM [22]. Generally, MRI and CT imaging are more commonly adopted to assess partial muscular components (PM); thus, PM took the major role in these diagnostic approaches, and PM-PM accounts for almost all the correlation pairs; there was only one GM-GM pair reported in the literature [22]. The superior correlation between MRI and CT could be attributed to the fact that they both measure muscular indexes at global or partial body scale; particularly, most studies have shown that when correlating the two parameters between MRI–CT, one parameter is usually a PM parameter.

MRI–US: Four of the 51 studies reported correlations of the derived parameters between these two diagnostic methods [28-31]. The average MRI–US correlation was reported as 0.861(SD=0.053) (Fig. 1B, Table 2B). The majority of the involved parameters in the MRI–US correlation studies were partial body muscular compositions: For MRI assessments, these included CSA (forearm, vastus lateralis (VL), rectus femoris (RF)) [28, 30, 31], muscle volume (VOL) of VL [30], and MT of RF and quadriceps [31]; while for US imaging, parameters are mainly MT (ulna, radius, VL, RF, and quadriceps) [28, 30, 31]. On the other hand, one research group analyzed the partial body fat composition in terms of mean fractional fat of a total leg for MRI and mean z-score of a total leg or RF for US. The Z-score could be calculated and converted to show the raw muscle echo intensities; thus, we considered the Z-score a muscle-related index [29] (Table 3). According to Figure 1C, the average correlation of PF-PM is 0.844 [29] and 0.868 for PM-PM [28, 30, 31]. The relatively high correlation of parameters between MRI and US may be partly attributed to the fact that the two correlating parameters were both measured at a partial body scale.

CT–US: Only three studies reported CT–US correlations [32-34]. The average CT–US correlation was reported as 0.875 (SD=0.039) (Fig. 1B, Table 4). Both CT and US tend to measure the partial index of muscle. CSA and the diameter of RF muscles are the only parameters used in the literature to derive the CT–US correlations. The highest correlation of 0.920 was reported from RF diameters in 45 older adults with cardiovascular diseases who participated in the sporting activities of a maintenance programme [33], followed by 0.880 and 0.826 from CSA of RF in a cohort with mixed healthy and COPD patients (n=18) [32], and a cohort of patients with chronic kidney disease (n=100) [34], respectively (Table 4). According to Figure 1C, the average correlation of PM-PM is 0.875 [32-34].

**Table 3:** The reported correlations of indexes derived between MRI and US.

Author	Year	Parameter MRI	Parameter US	Correlation (r)	Sample Size and Gender Distribution
**Abe et al.** [28]	2017	CSA of forearm(PM: Partial, Muscle)	MT of ulna(PM: Partial, Muscle)	0•937–0•946	10 subjects (8 males, 2 females)
		CSA of forearm(PM: Partial, Muscle)	MT of radius(PM: Partial, Muscle)	0•884–0•891	
**Mul et al.**[29]	2018	Mean fat fraction% of total legs(PF: Partial, Fat)	Mean z-score of total legs(PM: Partial, Muscle)	0.865	27 subjects (17 males, 10 females)
		Mean fat fraction% of RF(PF: Partial, Fat)	Mean z-score of RF(PM: Partial, Muscle)	right: 0.873	
				left: 0.794	
**Franchi et al.** [30]	2018	CSA of vastus lateralis (VL)(PM: Partial, Muscle)	MT of VL(PM: Partial, Muscle)	0.820	9 males
		Muscle Volume (VOL) of VL(PM: Partial, Muscle)	MT of VL(PM: Partial, Muscle)	0.760	
**Giles et al.** [31]	2014	MT of RF(PM: Partial, Muscle)	MT of RF(PM: Partial, Muscle)	0.858	5 subjects (2 males, 3 females)
		CSA of RF(PM: Partial, Muscle)	MT of RF(PM: Partial, Muscle)	0.897	
		MT of quadriceps(PM: Partial, Muscle)	MT of quadriceps(PM: Partial, Muscle)	0.915	

eviations: CSA: Cross-Sectional Area; MT: Muscle Thickness; VL: Vastus Lateralis; RF: Rectus Femoris; VOL: Muscle Volume

Overall, the correlations within IBMs tend to be higher for two possible reasons: Firstly, the IBM parameters are measured with similar body scales and/or components. Such homogeneous properties render relatively higher correlations. Secondly, MRI and CT are gold standards for sarcopenia assessment [8, 27] owing to their high-resolution feature for characterizing the properties of regional body compositions, which outperform DXA and BIA; and IBMs could reveal the characteristics of specific muscles for sarcopenia assessment in terms of volume, diameter, thickness, and CSA.

Notably, US excels in MRI and CT owing to its nature of being portable, radiation-free, and highly affordable. US also incorporates the advantages of MRI and CT of observing the severe degeneration, atrophy, training, or rehabilitation of a particular muscle and fat. Nonetheless, further studies are warranted to determine the cut-off points of MRI, CT, and US to inform sarcopenia conditions.

**Table 4:** The reported correlations of indexes derived between CT and US.

Author	Year	Parameter CT	Parameter US	Correlation (r)	Sample Size and Gender Distribution
**Seymour et al.** [32]	2009	CSA of RF(PM: Partial, Muscle)	CSA of RF(PM: Partial, Muscle)	0.880	56 subjects (27 males, 29 females)
**Thomaes et al.** [33]	2012	Diameter of RF(PM: Partial, Muscle)	Diameter of RF(PM: Partial, Muscle)	0.920	45 subjects (44 males, 1 female)
**Viviane et al.** [34]	2018	CSA of RF(PM: Partial, Muscle)	CSA of RF(PM: Partial, Muscle)	0.826	100 subjects (41 males, 59 females)

Abbreviations: CSA: Cross-Sectional Area; RF: Rectus Femoris

### 3.3. NIBM vs IBM

This review paper includes six NIBM–IBM pairs, including BIA–MRI, BIA–CT, BIA–US, DXA–MRI, DXA–CT, and DXA–US. The NIBM–IBM pairs are the most prevalent investigations, accounting for 31 articles out of the 51 included publications (Supplementary Table 5).

The Table 5-10 displays the reported correlations for various NIBM–IBM pairs from the literature. A summary of the correlations in the NIBM–IBM pairs is relatively complicated and challenging because NIBMs often measure whole-body composition, while IBMs generally assess partial muscle/fat-related indexes for sarcopenia assessments. Although MRI and CT can measure global body compositions, such applications are restricted by the high expenditure or potential radiation hazards. Hence, assessing the correlations between NIBMs and IBMs could be complex. Consequently, the reported correlations vary greatly (Fig. 1B, Table 5-10). For instance, the BIA–US pair yielded an average correlation of 0.463, while the DXA–MRI can achieve an average correlation of 0.834 (Fig. 1B). The complexity of various diagnostic techniques and measurement parameters in NIBM–IBM pairs may lead to lower correlation values than IBM–IBM and NIBM–NIBM (Fig. 1B). However, the lower correlations reported in NIBM–IBM pairs do not necessarily indicate the lack of efficiency in measuring sarcopenia.

**Figure 2.**
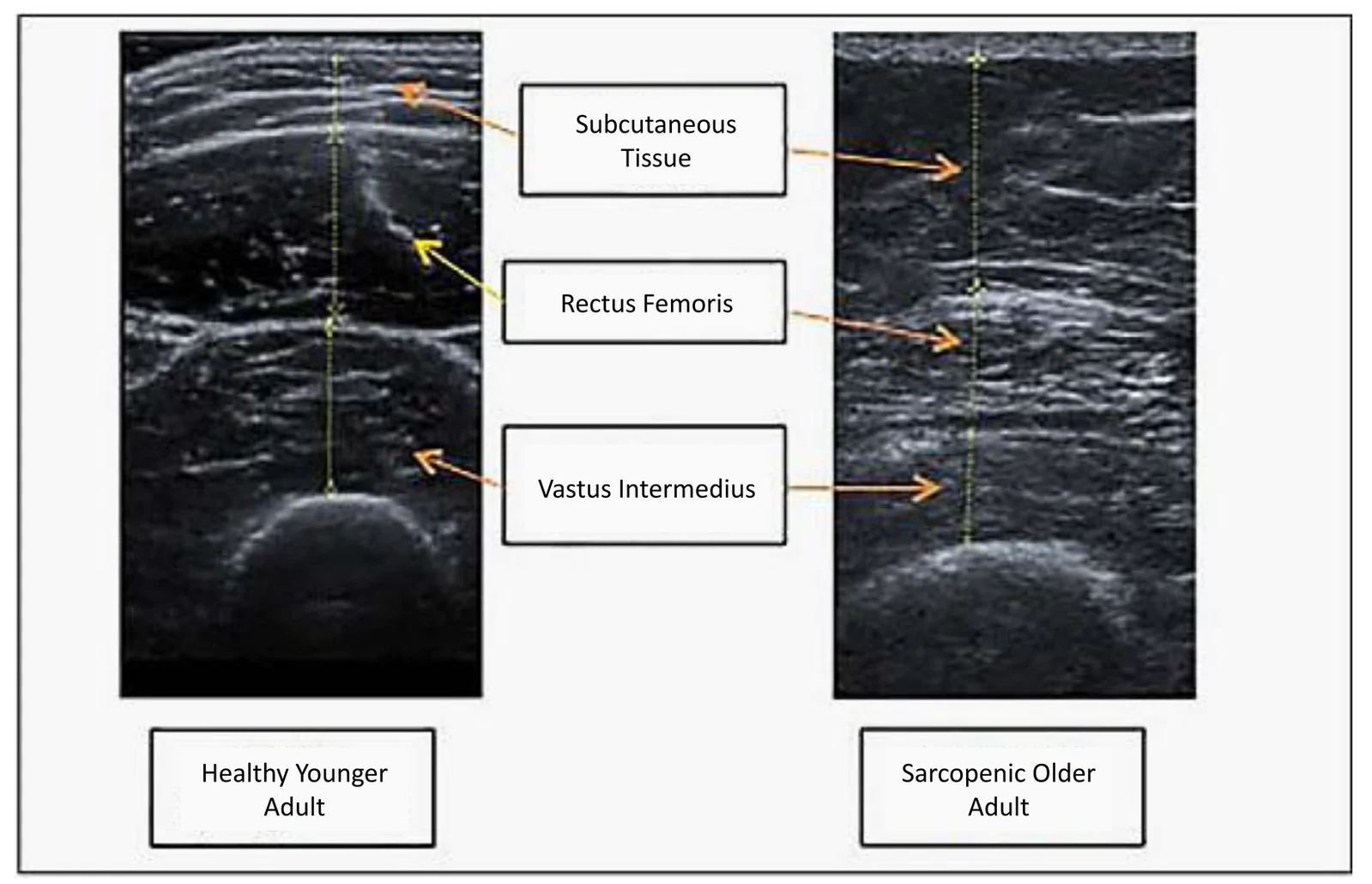
**The RF muscle under US for healthy younger adults and sarcopenic older adults (VI muscles, left is VL muscles, and right is VM muscles)** (adapted from Stringer et al., 2018 The Journal of Frailty & Aging [10])

BIA–US: Among the 51 included studies, seven investigated the correlations of sarcopenia-related indexes measured between BIA and the US imaging technique [35-41]. Table 5 shows that almost all of the measurement indexes from the US are “partial” body muscular compositions, while those from BIA are “global” body muscular/fatty compositions. For US, RF muscles are the ones primarily studied in the literature [35-41] for sarcopenia assessment because they are located down the thigh and serve the function of most daily human activities such as standing, running, sitting, and jumping [42]. Figure 2 illuminates the middle oval part of the RF muscle on a US image, where RF is oval and usually has a clear border; the upper part above the RF muscle is subcutaneous fat, while the vastus intermedius (VI) muscle, vastus lateralis (VL) muscle, and vastus medialis (VM) muscle, are located at the bottom, left, and right side of the RF, respectively.

The average correlation for this pair is 0.463 (SD=0.143) (Fig. 1B). As shown in Table 5, the highest correlation reported ranges from 0.685 to 0.698 between the CSA of RF from US and leg muscle mass/ASM/SMI from BIA in 58 older adults with haemodialysis [41]. Five literatures reported that correlations are lower than 0.500, among which, interestingly, almost all the measurement parameters from the US were the MT or CSA of thigh (quadriceps or rectus femoris) [35-37, 39, 40].

**Table 5:** The reported correlations of indexes derived between BIA and US.

Author	Year	Parameter BIA	Parameter US	Correlation (r)	Sample Size and Gender Distribution
**Kawai et al.** [35]	2017	PBF(Before control for age)(GF: Global, Fat)	MT of quadriceps(Before control for age)(PM: Partial, Muscle)	Men: 0.351 Women: 0.340	1,239 subjects (males: 511, females:728)
		SMI(Before control for age)(GM: Global, Muscle)	MT of quadriceps(Before control for age)(PM: Partial, Muscle)	Men: 0.454 Women: 0.414	
		PBF(After control for age)(GF: Global, Fat)	MT of quadriceps(After control for age)(PM: Partial, Muscle)	Men: 0.347 Women: 0.349	
		SMI(After control for age)(GM: Global, Muscle)	MT of quadriceps(After control for age)(PM: Partial, Muscle)	Men: 0.445 Women: 0.395	
**Hida et al.** [36]	2018	ASMI(GM: Global, Muscle)	MT of thigh(PM: Partial, Muscle)	0.380	201 subjects (males: 99 females: 102)
**Ramírez-Fuentes et al**. [37]	2019	FFM (kg)(G: Global)	CSA of RF(PM: Partial, Muscle)	COPD: 0.584 Healthy: 0.534	35 males
		FFM (% prediction.)(G: Global)		COPD: 0.606 Healthy: 0.494	
		Dry LM (kg)(G: Global)		COPD: 0.572 Healthy: 0.549	
**Wilkinson et al.** [38]	2020	ASM(GM: Global, Muscle)	CSA of RF(PM: Partial, Muscle)	0.650	113 subjects (43 males, 70 females)
		TMM(GM: Global, Muscle)		0.660	
**Battaglia et al.** [39]	2020	BCM(G: Global)	Top-quadriceps rectus femoris (QRFM) Thickness(PM: Partial, Muscle)	0.120	65 subjects (38 males, 27 females)
			Mid-QRFM Thickness(PM: Partial, Muscle)	0.258	
			Low-QRFM Thickness(PM: Partial, Muscle)	0.365	
			Abdominal subcutaneous fat tissue thickness(PF: Partial, Fat)	0.294	
**Simó-Servat et al.** [40]	2023	FM(GF: Global, Fat)	SF(PF: Partial, Fat)	Pre-surgical 0.420	32 subjects (8 males, 24 females)
				Post-surgical 0.520	
		SMI(GM: Global, Muscle)	TMT(PM: Partial, Muscle)	Pre-surgical 0.350	
				Post-surgical 0.380	
**Matsuzawa et al.**[41]	2021	Leg muscle mass(PM: Partial, Muscle)	CSA of RF(PM: Partial, Muscle)	0.685	58 subjects (36 males, 22 females)
		ASM(GM: Global, Muscle)		0.693	
		SMI(GM: Global, Muscle)		0.698	

Abbreviations: PBF: Percentage Body Fat; MT: Muscle Thickness; SMI: Skeletal Muscle Mass Index; ASM(I): Appendicular Skeletal Mass (Index); FFM: Fat-Free Mass; LM: Lean Mass; CSA: Cross-Sectional Area; TMT: Total Muscle Thickness; BCM: Body Cell Mass; FM: Fat Mass; TMT: Thigh Muscle Thickness; TMM: Total Muscle Mass; SF: Subcutaneous Fat; RF: Rectus Femoris; QRFM: Quadriceps Rectus Femoris

The relatively low correlations in BIA–US may be attributed to the fact that there existed a large variety in the measurement locations and components of the parameters between BIA and US. According to Figure 1C, the average correlation of G-PF is 0.294 [39], 0.358 for GF-PM [35], 0.454 for G-PM [37, 39], 0.470 for GF-PF [40], 0.502 for GM-PM [28, 29, 31, 33, 34], and 0.685 for PM-PM [34]. PBF and MT yielded a correlation of 0.340-0.454 in a study combining partial muscle with global fat [35]. It shows that the correlations between global and partial parameters, or between muscle-related and fat-related parameters, were generally lower (0.340-0.351 and 0.347-0.349, respectively), regardless of the subjects’ age. Furthermore, combining BIA and US for assessing the body composition in patients with specific diseases also showed good agreement [37, 38, 40, 41] (Table 5). For COPD subjects, a correlation of 0.584 was found between reduced CSA of RF muscle and FFM [37]. For patients with protein-energy wasting in haemodialysis, US detected a reduction in RF muscle, and the subcutaneous fat tissue thickness was weakly correlated (0.294) with BIA-generated parameters such as body cell mass (BCM) [39]. Although the correlation was small, with BCM correlations to the MT of top-RF (0.120), MR of maid-RF(0.258), MT of low-RF(0.365), and abdominal subcutaneous fat tissue thickness (0.294), the correlations were statistically significant with a p-value less than 0.05 [39]. Yet, it is worth noting that the cut-off points varied between the studies. For patients debilitated by CKD, the cut-off points were 8.9 cm2 for detecting sarcopenic muscle mass in males and 5.7 cm2 for females [38]. The correlation between the CSA of RF muscle from the US and BIA-generated ASM was 0.650 [38]. To sum up, GF-PM and G-PF led to the lowest correlation due to the high heterogeneity of the correlating parameters in terms of measurement locations and measurement components, making their correlations smaller than those in other sub-categories such as G-PM, GF-PF, GM-PM, and PM-PM. This may shed some light on the literature findings where the BIA–US category exhibited the lowest correlations with large variations among the ten pairs of comparisons (Fig. 1C). BIA–US measurements include various locations and components and combines the locations of both global and partial body with components of fat and muscle in a mixed manner; the complexity and diversity of the combinations lead to lower correlations in this category.

**Table 6:** The reported correlations of indexes derived between DXA and US.

Author	Year	Parameter US	Parameter DXA	Correlation (r)	Sample Size and Gender Distribution
**Berger et al.** [43]	2015	Total FFM (G: Global)	MT of right RF(PM: Partial, Muscle)	0.677	105 subjects (52 males, 53 females)
			MT of left RF(PM: Partial, Muscle)	0.691	
**Zhu et al.** [44]	2019	ALM(G: Global)	MT (anterior radial, anterior ulnar, posterior tibial, posterior fibula)(PM: Partial, Muscle)	Males: 0.248-0.539	265 subjects (97 males, 168 females)
				Females: 0.316-0.534	
**Álvarez et al.** [45]	2021	MT of the gastrocnemius medialis(PM: Partial, Muscle)	ALM of the gastrocnemius medialis(P: Partial)	Longitudinal plane: 0.689	57 subjects (24 males, 33 females)
				Transverse plane: 0.546	
**Souza et al.** [34]	2018	CSA of RF(using US and CT)(PM: Partial, Muscle)	LBM in the upper limbs(P: Partial)	0.286	100 subjects (41 males, 59 females)
			LBM in the lower limbs(P: Partial)	0.271	

Abbreviations: FFM: Fat-Free Mass; MT: Muscle Thickness; RF: Rectus Femoris; ALM: Appendicular Lean Mass; CSA: Cross-Sectional Area; LBM: Lean Body Mass

DXA–US: Among the 51 studies, four reported DXA–US correlations (Table 6) [34, 43-45]. The correlation in this category is relatively lower than the other categories, with an average of 0.497 (SD=0.166) (Fig. 1B). According to Figure 1C, DXA–US usually compares muscular components with the average correlation of P-PM, which is 0.448 [37, 38] and 0.547 for G-PM [43, 44]. Intriguingly, the highest correlation was reported between the global body fatty composition of the total FFM from the US and partial body muscular composition of the MT of RF muscle, ranging from 0.677 to 0.691 [43]. US may be regarded as a reliable and accurate alternative to DXA for sarcopenia assessment in older adults [43]. When measuring the MT of the anterior radial, anterior ulnar, posterior tibial, and posterior fibula with ALM, the correlation for males is less than for females [44]. Besides, the MT of the gastrocnemius medialis from the US and ALM of the gastrocnemius medialis from DXA resulted in the second highest correlation of 0.689 and 0.546 for the longitudinal and transverse planes, respectively [45]. Meanwhile, the reported correlations between the CSA of RF from the US and the Lean Body Mass (LBM) of upper/lower DXA were the lowest (range: 0.271-0.286) [34].

BIA–MRI: Five studies analyzed the correlation between BIA–MRI [46-50], with an average correlation of 0.715 (Fig. 1B, Table 7). Generally, BIA–MRI focused more on parameters measured at the global body scale, and both fat- and muscle-related components were distributed equally. According to Figure 1C, the highest correlation takes place in G-G comparison with correlation equaling 0.900 [48]. When BIA–MRI measures muscle- or fat-related indexes consistently from the global scale, the average correlation is higher than 0.7 (such as G-G, G-GM, GM-GM and GF-GF). When analyzing fat-related indexes between the global scale and partial scale, the average correlation of GF-PF is 0.622, which is smaller than the other combinations. Among the five studies, parameters with global body muscular/fatty compositions (GM/GF) yield higher correlations than parameters with partial body fatty compositions (PF) [46-50](Table 7). The highest correlation occurred when comparing visceral fat and total abdominal adipose tissue (VAAT), with a male of 0.940 and a female of 0.920[47]. However, a comparison between BIA-generated visceral fat and MRI-generated visceral adipose tissue area (AVAT) found a smaller correlation of 0.650 for males and 0.640 for females [47]. When global body fatty tissue was analyzed, it appears that the BIA-derived FFMM (BC) correlates highly with the derived FFMP (BC) with a correlation of 0.900. BFP from BIA and MRI also yield a high correlation of 0.900, respectively [48]; alternatively, visceral fat (VF) from BIA and total abdominal adipose tissue from MRI exhibited a higher correlation of 0.940 for males and 0.920 for females [47] (Table 7). However, it is worth noting that MRI-derived VF and BIA-derived VF exhibited lower correlations in two studies [46, 47].

**Table 7:** The reported correlations of indexes derived between BIA and MRI.

Author	Year	Parameter BIA	Parameter MRI	Correlation (r)	Sample Size and Gender Distribution
**Pietiläinen et al.** [46]	2012	Visceral Fat(Baseline) (GF: Global, Fat)	Visceral Index(Baseline) (GF: Global, Fat)	Females: 0.660	19 subjects (7 males, 12 females)
		Visceral Fat(5 months after the start of weight-loss intervention) (GF:Global, Fat)	Visceral Index(5 months after the start of weight-loss intervention) (GF:Global, Fat)	Sex-adjusted r= 0.777 Females: 0.810 Males: 0.780	
**Browning et al.** [47]	2012	Visceral Fat(GF: Global, Fat)	Visceral Adipose Tissue Area (AVAT) (PF: Partial, Fat)	Males: 0.650 Females: 0.640	120 subjects (60 males, 60 females)
		Visceral Fat(GF: Global, Fat)	Total abdominal adipose tissue (TAAT) (GF: Global, Fat)	Males: 0.940	
				Females: 0.920	
**Wang et al.** [48]	2013	Skeletal Muscle Percentage (SMP) (HBF BIA model) (GM: Global, Muscle)	SMP(GM: Global, Muscle)	0.850	200 subjects(100 males, 100 females)
		FFMP (BC) (G: Global)	FFMP(G: Global)	0.900	
		Visceral Fat Level (VFL) (HBF BIA model) (GF: Global, Fat)	VFL(GF: Global, Fat)	0.840	
		VFL (BC) (GF: Global, Fat)	VFL(GF: Global, Fat)	0.770	
		BFP (HBF BIA model) (GF: Global, Fat)	BFP(GF: Global, Fat)	0.890	
		BFP (BC) (GF: Global, Fat)	BFP(GF: Global, Fat)	0.900	
**Chaudry et al.** [49]	2020	Body Fat Mass (BFM) (GF: Global, Fat)	Volume of total adipose tissue(GF: Global, Fat)	Young: 0.680	63 males
				Old: 0.740	
			Fat fraction of the total abdominal volume of interest(PF: Partial, Fat)	Young: 0.760	
				Old: 0.570	
		Visceral Fat Area (VFA) (GF: Global, Fat)	Volume of the total adipose tissue(GF: Global, Fat)	Young: 0.640	
				Old: 0.700	
			Fat fraction of the total abdominal volume of interest(PF: Partial, Fat)	Young: 0.740	
				Old: 0.520	
			Volume of visceral adipose tissue(GF: Global, Fat)	Young: 0.490	
				Old: 0.490	
			Fat fraction of visceral volume of interest(PF: Partial, Fat)	Young: 0.680	
				Old: 0.390	
**Kiefer et al.** [50]	2022	Lean Body Mass Index (LBM) (G: Global) (Abdominal skeletal muscle mass index)	AMMI(GM: Global, Muscle)	0.704	335 subjects (188 males, 147 females)
		SMI(GM: Global, Muscle)	AMMI(GM: Global, Muscle)	0.581	

Skeletal Muscle Mass Index; TAAT: Total Abdominal Adipose Tissue; AVAT: Area of Visceral Adipose Tissue; BFM: Body Fat Mass; VFA: Visceral Fat Area; VFL: Visceral Fat Level; BC: BC532 (Tanita); SMP: Skeletal Muscle Percentage; HBF: HBF 359 (Omron)

Notably, weight-loss interventions could affect the correlation [46]. In the study of a 5-month weight-loss intervention, obese subjects improved the BIA–MRI correlation of VF from 0.660 to 0.810 in females, and for males, though the correlation was not significant at the baseline, it reached to 0.780 after five months of intervention. However, given the evidence that BIA could be inaccurate and could overestimate visceral fat, this may suggest that the weight-loss intervention might compensate for the overestimation of BIA measurement in obese subjects [48]. There was only one study investigating the BIA–MRI correlation of global body muscular compositions, where correlations ranged from 0.581-0.704 [50] (Table 7).

**Table 8:** The reported correlations of indexes derived between DXA and MRI.

Author	Year	Parameter DXA	Parameter MRI	Correlation (r)	Sample Size and Gender Distribution
**Chen et al.** [51]	2007	Whole body LSTM (G/P: Global/Partial)	Whole body SMM(GM/PM: Global/Partial, Muscle)	Whole body: 0.940	101 females
				Leg region: 0.910	
**Maden-Wilkinson et al.** [27]	2013	Thigh LM(P: Partial)	Thigh LM(P: Partial)	Young adult: 0.948	91 subjects:(45 males, 46 females)
				Older adult: 0.911	
**Yang et al.** [52]	2016	SF of thigh(PF: Partial, Fat)	BMI(G: Global)	0.907	190 subjects (58 males, 132 females)(Intermuscular Fat )
		IMF of thigh(PF: Partial, Fat)		0.963	
**Tavoian et al.** [53]	2019	Thigh LM(P: Partial)	Mid-thigh Muscle Volume (MV)(PM: Partial, Muscle)	0.890	26 subjects (10 males, 16 females)
		Thigh LM(P: Partial)	Mid-thigh MV(after 10 weeks)(PM: Partial, Muscle)	0.900	
**Brown et al.** [54]	2022	Whole-body SMM(GM: Global, Muscle)	Whole-body SMM(GM: Global, Muscle)	Males: 0.940	36 subjects (17 males, 19 females)
				Females: 0.940	
		Whole-body SMM(GM: Global, Muscle)	Single-slice thigh estimates of whole-body SMM(PM: Partial, Muscle)	Males: 0.880	
				Females: 0.870	
**Cho et al.** [55]	2022	ASM(GM: Global, Muscle)	MT of temporalis(PM: Partial, Muscle)	0.379	68 subjects (11 males, 57 females)
		BMI(G: Global)		0.303	

Abbreviations: LSTM: Lean Soft Tissue Mass; SMM: Skeletal Muscle Mass; LM: Lean Mass; SF: Subcutaneous Fat; BMI: Body Mass Index; SMM: Skeletal Muscle Mass; ASM: Appendicular Skeletal Mass; BMI: Body Mass Index; MT: Muscle Thickness; MV: Muscle Volume; IMF: Intermuscular Fat

DXA–MRI: Six studies analyzed the correlation between DXA–MRI [27, 51-55], with an average correlation of 0.834 (Fig. 1B, Table 8). Intriguingly, the greatest correlation of 0.948 was reported for partial thigh LM [27], followed by the correlation of 0.940 between the whole-body SMM and whole-body Lean Soft Tissue Mass (LSTM) [51], and the correlation of 0.940 for SMM [54] (Table 8). Alternatively, when partial body fatty tissue was analyzed, intramuscular fat (IMF) of the thigh and BMI achieved the highest correlation of 0.963; meanwhile DXA-derived Subcutaneous Fat (SF) of the thigh achieved a correlation of 0.907 with BMI [52] (Table 8). Of note, one study focused on older female adults with probable Alzheimer’s disease (without weakness) and reported a remarkably low correlation of MRI-derived partial temporalis MT with DXA-derived global ASM (0.379) and DXA-derived BMI (0.303) [55] (Table 8). In Figure 1C, for DXA–MRI, when analyzing the correlations of muscle-related parameters on the global scale and partial scale (GM-PM), the average correlation was 0.710; whilst when analyzing correlations of partial composition with one another (P-PM, P-P, PF-G, G-GM, GM-GM), the average correlation was above 0.9, especially for muscle-related indexes. However, when analyzing compositions from different body locations, such as G-PM, the correlation appeared to be the smallest, with an average of 0.303. Intriguingly, we noted that MRI-derived parameters generally generated greater correlations with parameters measured from other diagnostic approaches, such as in the case of MRI–CT (average=0.978), MRI–US (average=0.861), BIA–MRI (average=0.715), and DXA–MRI (average=0.834) (Fig. 1A&1B)

**Table 9:** The reported correlations of indexes derived between BIA and CT.

Author	Year	Parameter BIA	Parameter CT	Correlation (r)	Sample Size and Gender Distribution
**Gibson et al.** [56]	2014	FFM(G: Global)	FFM(G: Global)	Colorectal cancer: 0.795	43 patients (27 males, 16 females)
		FFMI(G: Global)	FFMI(G: Global)	Colorectal cancer: 0.585	
**Jo et al.**[57]	2018	ASM(GM: Global, Muscle)	LSMA(PM: Partial, Muscle)	Males: 0.724	1,162 subjects(641 males, 521 females)
				Females: 0.645	
		ASM(GM: Global, Muscle)	LSMA(PM: Partial, Muscle)	0.898	
		ASM(BMI adjusted)(GM: Global, Muscle)	LSMA(BMI adjusted)(PM: Partial, Muscle)	0.858	
**Ohara et al.** [58]	2020	SMI(GM: Global, Muscle)	SMI(GM: Global, Muscle)	0.610	110 subjects (71 males, 39 females)
		SMI(>6 months post-intervention for CLD)(GM: Global, Muscle)	SMI(>6 months post-intervention for CLD)(GM: Global, Muscle)	0.510	
**Grossberg et al.** [59]	2021	SM mass(GM: Global, Muscle)	SM CSA(lumbar region)(PM: Partial, Muscle)	Head & neck cancer: 0.969	48 subjects (40 males and 8 females)
		FFM(G: Global)	SM CSA(lumbar region)(PM: Partial, Muscle)	Head & neck cancer: 0.969	
		FM(GF:Global, Fat)	Adipose CSA(lumbar region)(PF: Partial, Fat)	Head & neck cancer: 0.948	
		SMI(GM: Global, Muscle)	SMI(lumbar region)(PM: Partial, Muscle)	Head & neck cancer: 0.948	
		FFMI(G: Global)	SMI(lumbar region)(PM: Partial, Muscle)	Head & neck cancer: 0.927	
		FMI(GF: Global, Fat)	(Adipose Index) ADI(lumbar region)(PF: Partial, Fat)	Head & neck cancer: 0.954	
**Cao et al.** [60]	2022	BMI(Observed BIA)(G: Global)	Predicted BMI(Erector spinae muscle area)(P: Partial)	0.935	606 subjects (384 males, 222 females)
		BMI(G: Global)	SMM(PM: Partial, Muscle)	0.897	
**Looijaard et al.** [61]	2020	MM(*Talluri)(GM: Global, Muscle)	MM at L3 level(PM: Partial, Muscle)	0.834	110 subjects (75 males, 35 females)

Abbreviations: FFM: Fat-Free Mass; FFMI: Fat-Free Mass Index; ASM: Appendicular Skeletal Mass; LSMA: Lumbar Skeletal Mass Area; SMI: Skeletal Muscle Mass Index; SM: Skeletal Muscle; CSA: Cross-Sectional Area; SMI: Skeletal Muscle Mass Index; ADI: Adipose Index; BMI: Body Mass Index; MM: Muscle Mass; CLD:chronic liver disease; SMM: Skeletal Muscle Mass

(*Talluri who generated the equation for calculating skeletal muscle mass)

BIA–CT: Six studies analyzed the BIA–CT correlation [56-61], with an average correlation of 0.824 (SD=0.147) (Fig. 1B, Table 9). In table 9, the parameters of BIA measurement are ASM, ALMM, FFM, and SMI. The parameters of CT measurement are BMI, SMI, SM CSA, fat-related CSA, and lumbar skeletal mass area (LSMA). Generally, the highest correlation was for the head and neck cancer patients’ diagnosis of body compositions, correlation between SM mass and SM CSA is 0.969, between FFM and SM CSA is 0.969, between FM and Adipose CSA is 0.948, between SMI and SMI is 0.948, between fat-free mass index (FFMI) and SMI is 0.927, and between fat mass index (FMI) and ADI is 0.954 [59] (Table 9). According to Figure 1C, the correlations of fat-related indexes such as GF-PF are higher than those of muscle-related indexes in the same context such as GM-GM and GM-PM[58, 59, 61].

**Table 10:** The reported correlations of indexes derived between DXA and CT.

Author	Year	Parameter DXA	Parameter CT	Correlation (r)	Sample Size and Gender Distribution
**Bredella et al.** [62]	2010	Trunk Fat Mass(PF: Partial, Fat)	Abdominal fat area(PF: Partial, Fat)	Obese: 0.940	91 females
				Lean: 0.930	
		Leg Fat Mass(PF: Partial, Fat)	Thigh fat area(PF: Partial, Fat)	Obese: 0.940	
				Lean: 0.940	
		Leg LSTM(P: Partial)	Thigh muscle area(PM: Partial, Muscle)	Obese: 0.760	
				Lean: 0.770	
**Kim et al.** [63]	2023	ASMI(GM: Global, Muscle)	ASMI(GM: Global, Muscle)	0.794	120 subjects (77 males, 43 females)
**Yoo et al.** [64]	2022	Total soft tissue mass of thigh(P: Partial)	Soft tissue mass(G/P: Global/Partial)	Whole: 0.909	100 subjects (40 males, 60 females)
				Thigh: 0.973	
**Tsukasaki et al.**[65]	2020	SMI(GM: Global, Muscle)	CSA of mid-thigh(PM: Partial, Muscle)	Men: 0.800	1818 subjects (943 males, 875 females)
				Women: 0.710	
		CSA of the quadriceps(PM: Partial, Muscle)	Men: 0.750	Women: 0.630	

Abbreviations: ASMI: Appendicular Skeletal Mass Index; SMI: Skeletal Muscle Mass Index; CSA: Cross-Sectional Area

DXA–CT: Four studies analyzed the DXA–CT correlation [62-65]with an average correlation of 0.834 (SD=0.105) (Fig. 1B, Table 10). Generally, DXA–CT correlations were high (>0.900) for partial body compositions between DXA-derived total soft tissue mass of the thigh and CT-derived thigh soft tissue mass (0.973) [64]; correlations are also high for DXA-derived trunk fat mass and CT-derived abdominal fat area (obese subjects: 0.940; lean subjects: 0.930), and DXA-derived leg fat mass and CT-derived thigh fat area (0.940 for both obese and lean subjects [62]). Remarkably, the DXA-derived partial leg LSTM exhibited lower correlations with CT-derived thigh muscle area (obese subjects: 0.760; lean subjects: 0.770), which is lower than the correlation of partial fat-related indexes (Table 10)[62]. On the other hand, lower correlations were reported for DXA-derived global muscular SMI with CT-derived partial cross-sectional muscle area of the mid-thigh (males: 0.800; females: 0.710) and with CT-derived partial CSA of the quadriceps (males: 0.750; females: 0.630) [65]. Notably, only one study reported DXA–CT correlations with GM-GM parameters, global ASMI, achieving a correlation of 0.794 (Table 10) [63]. According to Figure 1C, correlations of similar indexes such as PF-PF achieved the highest correlation, whereas dissimilar indexes such as GM-PM yielded smallest correlation.

To sum up, the NIBM–IBM category incorporates a wide variety of combinations of measurement parameters (particularly in terms of the heterogeneity of the measurement locations and components between the two methods), compared to the NIBM–NIBM and IBM–IBM. Such a heterogeneity leads to lower correlations reported for NIBM–IBM. According to Figure 1C, the most common combinations are PM-PM, GM-GM, P-P, or G-G for IBM–IBM and NIBM–NIBM. In stark contrast, the variety of combinations expanded to GM-PF, P-GM, G-PM, G-PF, and P-GF in NIBM–IBM.

## 4. DISCUSSION

Sarcopenia is a debilitating disease affecting countless older adults worldwide. Early diagnosis allows for timely interventions to avoid further worsening of the sarcopenia conditions [66-72]. Modern diagnostic approaches include CT, MRI, US, DXA, and BIA, categorized into the IBMs and NIBMs in this review article. Numerous studies have been conducted to investigate the correlations of measurement parameters among these five technologies, and this review article is the first to systematically review the overall findings among all these five approaches, with overarching goals to enhance the understanding of this topic and provide valuable insights and recommendations for future research.

**Table 11:** Framework of NIBM–IBM selections.

Combination	Portability	High Costs	High Time Efficiency	High Risks of Radiation Exposure	Examples of Common Parameters (Scales)
BIA–MRI	×	√	×	×	BIA: Visceral Fat, SMP, BFP, SMI (include global fat and muscle parameters) MRI: Visceral Adipose, ASMM (include global fat and muscle scale)
BIA–CT	×	√	×	√	BIA: FFM, ASM, SMI, FM, SMI (include global muscle and global fat scale) CT: FFM, LSMA, SMI, CSA (include partial muscle, global muscle, and partial fat scale)
BIA–US	√	×	√	×	BIA: PBF, SMI, ASMI, FFM, FM (include global fat and muscle parameters) US: MT, CSA, Abdominal subcutaneous fat tissue thickness (include partial muscle and partial fat scale)
DXA–MRI	×	√	×	√	DXA: Whole-body LSTM, Thigh LM, Whole-body SMM (include global muscle, partial muscle, and partial fat scale) MRI: Whole-body SMM, Thigh LM, BMI, MV, and MT (include global muscle and partial muscle scale)
DXA–CT	×	√	×	√	DXA: FM, LSTM, ASMI, SMI (include partial fat and global muscle scale) CT: Abdominal fat area, thigh muscle area, CSA, ASMI (include partial fat, global muscle, and partial muscle scale)
DXA–US	×	√	×	√	DXA: MT, ALM (include partial muscle scale) US: MT and CSA (partial muscle scale)

Abbreviations: SMP: Skeletal Muscle Percentage; BFP: Body Fat Percentage; SMI: Skeletal Muscle Mass Index; ASMM: Appendicular Skeletal Muscle Mass; FFM: Fat-Free Mass; ASM(I): Appendicular Skeletal Mass (Index); FM: Fat Mass; SMI: Skeletal Muscle Mass Index; LSMA: Lumbar Skeletal Mass Area; CSA: Cross-Sectional Area; PBF: Percentage Body Fat; MT: Muscle Thickness; LSTM:Lean Soft Tissue Mass; LM: Lean Mass; SMM: Skeletal Muscle Mass; BMI: Body Mass Index; MV: Muscle Volume; ALM: Appendicular Lean Mass

### 4.1 Understanding the Correlations

The findings of this review demonstrate that the IBM–IBM correlations and NIBM–NIBM correlations were generally higher compared to the NIBM–IBM correlations. The lower correlation values reported for NIBM–IBM may be largely attributed to the heterogeneous measurement locations and components between the compared diagnostic methods (Fig. 1C, Table 3A-F). For instance, in NIBM, correlation analyses were mostly conducted to compare either the global or partial indexes between the two compared diagnostic methods (Fig. 1C, Table 1); similarly, in IBM, analyses were typically performed to compare partial indexes between the diagnostic methods (Fig. 1C, Table 2A-C).

Given the context of similar tissue components and measurement scopes, the IBM–IBM and NIBM–NIBM correlations are generally higher than the NIBM–IBM correlations. Meanwhile, the correlations between similar measurement components of the diagnostic methods (e.g., muscle-to-muscle or fat-to-fat) tend to be higher than those between dissimilar components (e.g., muscle-to-fat). The correlations of GF-GF, GM-GM, PF-PF, and PM-PM were remarkably higher (Fig. 1C). Since the “global” scale measures both the muscle-related components and indexes of soft tissue, FFM, LM, and indexes incorporating even body water and bone mass, it is important to involve “global” indexes in the sarcopenia evaluation matrix to obtain more systemic and representative information concerning the body components of interest. However, incorporating more indexes measured at different body locations or scales into the correlation comparison could lead to a lower correlation. If the measurement site is different or the measurement composition is different, it tends to produce a lower correlation compared to conditions where the correlations were made between similar sites and compositions.

According to AWGS, EWGSOP, and IWGS, the major parameters related to sarcopenia are the appendicular skeletal muscle index (ASMI) and appendicular skeletal muscle mass (ASMM). We also include skeletal muscle mass (ASM), muscle mass (MM), lumbar skeletal muscle mass area (LSMA), abdominal skeletal muscle mass (AMMI), and other indexes that relate to muscles and sarcopenia in Supplementary Table 9. The correlations of strongly correlated sarcopenia factors show a similar trend, where comparisons within NIBM or IBM (such as NIBM–NIBM and IBM–IBM) exhibit a higher correlation compared to NIBM–IBM. By contrast, the correlation between NIBM and IBM is lower. Lower correlations exist within a mixture of measurement aspects, such as the correlations between globally measured fat-related parameters with partially measured muscle-related parameters, as in the case of BIA–US (Table 3A) [35]. For example, in the study of Kawai, H., et al. [35], the correlations are 0.351 for men and 0.340 for women when comparing BIA and US. The parameter BIA provided is PBF, which is a global fat (GF) index, and the parameter US provided is MT, which is a partial muscle (PM) index. Thus, the correlation is relatively lower. In the study by Zwart, A.T., et al. [22], the correlation is 0.997 when both MRI and CT provide a similar global muscle (GM) index, as measured by SMI for comparison. Another example is that in the study of Franchi, M. V., et al. [30], the correlation was 0.820 when MRI provided the CSA of the vastus lateralis and US provided the MT of the vastus lateralis, and though CSA and MT are different parameters, the correlation is relatively high since both CSA and MT measure a partial muscle (PM) index. The correlations between the two approaches should be interpreted cautiously, considering the underlying measurement location and components.

### 4.2 Inaccuracy within the Approaches and Obesity

Notably, several studies have reported that BIA and DXA can over- or underestimate the measured values, thereby causing inaccuracies in subsequent correlation analyses. For example, in the BIA–DXA study, Yi et al. showed that HF-BIA underestimated ALM and PBF, whereas it overestimated FFM [15]. Cruz Rivera et al. concluded that BIA underestimated the fat mass and PBF [17]. In light of this, several research groups have adopted equations after adjustments for BIA-derived ALM and have achieved a higher correlation [19, 20]. Apart from this, for certain diseases, such as type 2 diabetes, the overestimation of BIA can result in a lower correlation compared to normal controls [18]. In DXA–MRI studies, Maden-Wilkinson et al. reported that DXA underestimated the age-related loss of thigh muscle mass and thus produced a lower correlation with MRI[27]. Similarly, in DXA–CT studies, Bredella et al. stated that DXA underestimated the trunk and thigh fat but overestimated the thigh muscle mass for obese women [62].

Sarcopenia obesity (SO) also tends to cause inaccurate results via BIA and DXA. SO refers to a condition of older people with reduced muscle mass but a higher level of adiposity, and it is also associated with cardiovascular disease (CVD). It can increase the risks of mortality[73-76]. Extensive research suggests that it is important to evaluate the muscle and fat mass in SO [71]. Obesity can lead to inaccuracies in BIA measurements; for example, BIA may overestimate visceral fat in males [46]. Although fat and obesity can cause inaccuracy in NIBM measurements, several research groups have demonstrated that NIBM performs well for specific measurements. For example, BIA provided accurate results when measuring changes in FM and skeletal muscle-related parameters, despite the inaccuracy of fat-related parameters, such as visceral fat [46]. BIA underestimated FM and overestimated FFM for subjects with a greater BMI over 34 kg/m2 [77]. In the BIA–MRI comparison, interference in weight loss may compensate for the overestimation of BIA in obese people[41]. On the other hand, DXA overestimated the thigh lean mass by 10%, LMM by 47%, and TFM by 20% compared to DXA–CT [64]. The underestimated trunk and overestimated thigh muscle mass from DXA could also affect the results of the correlation analyses, and the errors may increase with increasing body weight [41, 46, 76]. Therefore, we speculated that the above measurement bias using DXA and BIA may be partly attributed to their relatively lower correlations with other diagnostic techniques, which are more accurate. To this end, we urge attention when considering the correlations with BIA or DXA, which should be processed cautiously, especially when handling parameters that can be influenced by obesity.

### 4.3 Implications for Clinical Practice

Although drawing a conclusion about which pair is the most effective for measuring sarcopenia is beyond the scope of this review, we recommend combining the IBM and NIBM methods for sarcopenia assessment because they may work in tandem to leverage the strengths of each and measure complementary metrics, thereby providing a more holistic understanding of sarcopenia. Considering the capability, affordability, and scope of applicability, the greatest potential lies among NIBM–IBM combinations.

To measure sarcopenia comprehensively, it is important to consider the body conditions on two scales: whether the index is measured at a holistic or partial body scale, and whether the index indicates muscular or fat-related conditions. NIBM and IBM are recommended to operate jointly and be complementary to each other, providing measurements based on the above two scales. In light of this, choosing one technique from NIBM (BIA and DXA) can generate a global parameter, while another from IBM (MRI, CT, and US) can provide a partial parameter. In total, this review presents six pairs of NIBM–IBM (BIA–MRI, BIA–CT, BIA–US, DXA–MRI, DXA–CT, DXA–US). The principle for IBM and NIBM selections lies in the objective and purpose of measurement. For instance, when selecting IBM, if the goal is to investigate a specific aetiology or pathology that requires an MRI or CT scan, sarcopenia-related indicators can be measured simultaneously. However, suppose the sole purpose is to evaluate sarcopenia. In that case, we recommend ultrasound (US) as it is more convenient, with no risk of ionizing radiation, and cost-effective, making it suitable for repeated routine examinations. When selecting NIBMs, DXA may be chosen if there is a need to measure bone density. Otherwise, BIA is more convenient to operate and more affordable, especially for home and community-based screening.

The combination of DXA–CT, for instance, can measure the holistic body composition, such as LM, while also assessing localized MT, such as in the thigh. Similarly, the combination of BIA and MRI can evaluate BFP while providing detailed measurements of MV. However, it is noticeable that MRI and CT are not suitable for routine muscle screening due to their high cost and operational complexity, as well as the risks associated with CT-induced radiation. As for the combination of DXA–MRI, although both DXA and MRI generate detailed and precise results, the examination can be expensive and is therefore not recommended for daily routine assessment. MRI is not suitable for individuals wearing metallic devices, and it is bulky and not as available as US in some rural areas. Therefore, the US is recommended for assessing partial muscle conditions. On the other hand, although DXA allows for measurements of the global body index, it involves low-dose radiation and is not practical for in-home or community routine screening compared to BIA. In stark contrast, BIA becomes suitable for routine body composition monitoring by ensuring the patient's condition during measurements, and its accuracy has been improved along with technological advancements. As for the US, although it requires trained professionals to perform scans, and biases may exist among different operators, healthcare providers can be trained to conduct these operations professionally. Compared to BIA–US, BIA–CT, and DXA–US, these methods can be used when there is a need for specific CT scanning or DXA scanning, as prescribed by a medical doctor, to avoid exposing patients to unnecessary radiation for sarcopenia screening.

Among the six pairs, we recommend the BIA–US combination, as it presents distinct advantages. First, from the perspective of the scope of use, BIA–US enables the assessments of different tissues (muscle and fat) and at different body scales (globally and partially), which may provide complementary information for sarcopenia assessment. For instance, BIA–US generates G (FFM, BCM), GM (SMI, ASMI, ASM, TMM), GF (PBF, FM), PM (leg muscle mass, TMT, MT, CSA), and PF (SF, abdominal subcutaneous fat tissue thickness) metrics. The combination of them provides a large variety of measurement components at both partial and global body scales, offering a more holistic picture of the sarcopenia condition. Combining US and BIA enables the detection of both muscle- and fat-related parameters globally and partially. This combination performs collaboratively in providing a more applicable and comprehensive picture of the risk or severity of sarcopenia. Second, from the perspective of safety, BIA–US favours frequent, repeated, portable, and even population-wide assessments without the risk of potential radiation hazards, which would otherwise be impractical for other technologies, such as CT and DXA. Third, from the perspective of applicability and affordability, BIA–US is portable and of greater affordability compared to other technologies (such as MRI, CT, X-ray, etc.); US can also generate real-time results and observe muscle activity of contraction. These properties would make BIA–US a portable, combination assessment tool suitable for use in clinics, the home, and community settings. Table 4 shows the framework of NIBM–IBM selections.

Given the heterogeneous measurement locations and components of these two diagnostic approaches, the lower averaged correlations may not necessarily represent their low clinical value for sarcopenia assessments. Indeed, they may be complementary to each other and hence play a concerted role in reflecting sarcopenia. The area of research on BIA–US for sarcopenia assessment deserves a great deal of attention for further investigations, and more solid evidence on this emerging approach is highly anticipated, with the hope of revolutionizing novel and holistic strategies for sarcopenia assessments in the long run.

### 4.4 Limitations in the potential biases

This review article presents several limitations, particularly given the presence of biases of reporting the sample size, population heterogeneity, and study methodologies. Firstly, from the gender distribution aspect, overrepresentation or underrepresentation of one gender may hinder the generalization of the findings to general populations. Some pathological characteristics may vary by gender, and uneven distribution could obscure the results. For example, according to Supplementary Figure 1, some pairs involve more males than females, such as MRI–US and MRI–CT (male-to-female: ~7:3), and others involve more females than males, such as DXA–MRI (male-to-female: ~3:7). Secondly, from the sample size aspect, the sample sizes are different in each subcategory, thus potentially introducing bias into the results. For example, BIA–DXA entails the largest number of subjects in regard to sample size (n=4194), while MRI–US involves a sample size of 51, as illuminated in Supplementary Figure 1. Thirdly, from the population aspect, the diversity in ethnicity and culture could also influence the reliability of the results. As summarized in Supplementary Table 5, among the 51 included studies, only three studies conducted by Battaglia, Y et al. [39], Pietiläinen KH, et al. [46], and Chen Zhao et al. [51] explicitly reported the ethnicity of the subjects, while others did not provide such information. Thus, whether or not and how this variation between the studies would introduce bias to our findings requires further investigation. Therefore, the readers of this review article are recommended to cautiously interpret the reported findings. In future research, studies that are dedicatedly designed to mitigate the abovementioned potential impacts are greatly anticipated.

### 4.5 Future Direction and Limitations

Firstly, although we attempted to include as many related publications from 2007 as possible up to February 2023, resulting in a total of 51 studies, more recent studies might not be included at the time that this manuscript was prepared. Secondly, this review classified the approaches by whether the result is generated from images. Further classification techniques are required for a more thorough investigation. For example, we could classify the groups by age, gender, disease group, community, or clinical setting. Thirdly, the amount of some correlational studies is less than others; for example, there are only three correlation studies for CT–US and DXA–US. We encourage more studies in these two fields to show how measuring locations and components could affect the correlations in between them. Fourthly, software for acquiring and analyzing the measurement data embedded in each of the diagnostic approaches would affect the derived result. Thus, further investigation is needed to measure the accuracy of software updates. Lastly, we acknowledge that it is of more interest to reveal the most effective diagnostic approaches or measurement locations/components for sarcopenia assessments, but reaching a conclusion on this topic appears to be beyond the scope of this review article. We hope that the findings of this review will provide the community with enhanced understanding and insights for future research to determine an ideal evaluation approach and the parameters for sarcopenia measurement.

## 5 Conclusion

Sarcopenia is a crippling disease affecting older adults worldwide, and it was internationally introduced into the ICD-10 code. Modern technologies for sarcopenia assessment include BIA, DXA, MRI, CT, and US. It is essential to note that higher correlations occur within the same category (i.e., IBM vs. IBM and NIBM vs. NIBM) and with parameters that share similar measurement locations and components. In stark contrast, the correlations between parameters with dissimilar measurement locations and components tend to be lower, as in the NIBM–IBM category. However, the relatively low NIBM–IBM correlations do not necessarily represent their low clinical value for sarcopenia assessments due to the huge heterogeneity of the measurement locations and components in the current literature. Indeed, they may be complementary to each other and hence provide a more comprehensive picture of the risk or severity of sarcopenia. To this end, the BIA–US category exhibits an exciting direction and should deserve a great deal of attention for future investigations, particularly given the US capabilities in estimating both muscle quantity and quality and assessing the fat-related parameters as well as its untapped potential following its recent introduction for sarcopenia assessments in 2018 by the SARCUS working group, integrating US with BIA is an exciting direction for a more holistic, safe, and low-cost bedside sarcopenia assessment in the future.

## Supplementary Materials

The Supplementary data can be found online at: www.aginganddisease.org/EN/10.14336/AD.2025.0145.
