# Supplementary material for: A Comprehensive Review of the Correlations of Measurement Parameters among Modern Technologies for Sarcopenia Assessment
Source: Aging Dis. 2025 May 8;17(3):1423–45. doi: 10.14336/AD.2025.0145 (PMC13061567; doi:10.14336/AD.2025.0145)
Supplement: Supplementary file 1 — The Supplementary data can be found online at: www.aginganddisease.org/EN/10.14336/AD.2025.0145. [file AD-17-3-1423-s.pdf]

A Comprehensive Review of the Correlations of Measurement Parameters among Modern Technologies for Sarcopenia Assessment

Dawei Zhang, Sai Kit Lam, Yongping Zheng

Table 1. Criteria of AWGS Consensus of Sarcopenia

AWGS	2014[78]	2019[3]
**Description**	1) low muscle mass<br>2) low muscle strength<br>3) low physical performance	
**People**	>60 or 65 years old, community dwelling	>60 or 65 years old, community dwelling and **hospital settings**
**DXA**	ASMI:<br>men: <7.0 kg/m^2<br>women: <5.4 kg/m^2	ASMI:<br>men: <7.0 kg/m^2<br>women: <5.4 kg/m^2
**BIA**	ASMI:<br>men: <7.0 kg/m^2<br>women: <5.7 kg/m^2	ASMI:<br>men: <7.0 kg/m^2<br>women: <5.7 kg/m^2
**Hand Gripping**	men: <26 kg<br>women: <18 kg	**men: <28 kg**<br>women: <18 kg
**Gait Speed**	0.8 m/s	
**6-Metre Walk**	1.0 m/s	
**SPPB Score**	<= 9	
**5-Time Chair Stand**	>12 seconds	
**Calf Circumference**	men: 34 cm<br>women: 33 cm	
**SARC-F**	>=4	
**SARF-Calf**	>=11	

Table 2. Criteria of EWGSOP Consensus of Sarcopenia

EWGSOP	2010[79]	2019[12]
**Description**	1) low muscle strength<br>2) low muscle quantity or quality<br>3) low physical performance	
**People**	>65 years old	
**DXA**	ASMI:<br>Men: <7.26 kg/m^2<br>Women: <5.45 kg/m^2	ASMM:<br>Men: <20 kg<br>Women: <15 kg<br>ASMI:<br>Men: 7.0 kg/m^2<br>Women: 5.5 kg/m2
**BIA**	ASMI: (Pre-sarcopenia)<br>Men: 8.51-10.76 kg/m^2<br>Women: 5.76-6.76 kg/m^2<br>ASMI: (Sarcopenia)<br>Men: <=8.50 kg/m^2<br>Women: <=5.75 kg/m^2	
**Hand Gripping**	Men: < 30 kg<br>Women: < 20 kg	Men: < 27 kg<br>Women: < 16 kg
**Gait Speed**	0.8 m/s	
**TUG**	>= 20 s	
**SPPB Score**	<= 8	
**5-Time Chair Stand**	>15 seconds	
**400-metre walk test**	>=6 min or non-completion	

Table 3. Criteria of IWGS Consensus of Sarcopenia

IWGS	2011[13]
**Description**	Loss of skeletal muscle mass and function
**People**	Aged Group
**ASMI**	Men: 7.23 kg/m^2<br>Women: 5.67 kg/m^2
**Gait Speed**	1 m/s

Table 4. Pros and Cons in BIA/DXA/MRI/CT/US

Approach	Pros	Cons
**BIA [14,80,81,82,83,84,85]**	• Low cost<br>• non-invasive<br>• portable<br>• radiation-free<br>• capability of offering immediate assessment results	• inaccuracy due to obesity, temperature, and hydration of the body<br>• incapability in measuring a site-specific component
**DXA [9,86,87,88,89]**	• Able to measure bone-related parameters	• radiation-carrying (though small)<br>• device is bulky, portable<br>• accuracy due to obesity
**MRI [90,91,92]**	• high-resolution<br>• radiation-free<br>• precise and able to describe changes appearing in the intracellular environment<br>• non-invasive	• expensive<br>• not eligible for subjects with metal supplements worn or implanted devices<br>• not for patients with claustrophobia<br>• non-portable
**CT [88,93,94]**	• high-resolution<br>• precise<br>• has been used as a first-line diagnostic modality for various diseases, thus could measure sarcopenia and other diseases at a time	• radiation exposure<br>• expensive<br>• demand for professional training of operators with licensing in operating radiation-barring equipment<br>• non-portable
**US [95,96,97,98,99,100]**	• radiation-free<br>• highly accessible<br>• real-time<br>• low-cost<br>• portable	• reliance on professional training for image scanning<br>• not able to scan for whole-body (global) index

Table 5. Demographics Table
(Italicized studies contained subject age less than 40)

Author	Year	Sample Information	Brand	Age Information	Setting	Healthy Status	Statistically Approach	Category
**NIBM-NIBM**								
Yi et al. [15]	*2022*	*109 people (50.5% male)*	*BIA: Inbody 970*<br>*DXA: Lunar Prodigy*	*male age:43.4 ± 14.7 years old*<br>*female age: 44.9 ± 14.1 years old*	*enrolled via public advertisements*	*could stand alone for over five minutes without assistance*	*Pearson's*	BIA-DXA
			BIA: BWA (clamp)<br>*DXA: Lunar Prodigy*					
			BIA: BWA (adhesive)<br>*DXA: Lunar Prodigy*					
Cruz Rivera et al.[17]	2022	50 COPD Veterans (96% male)	BIA: Omron HBF-306C<br>DXA: GE Lunar, Madison	mean age: 69.5 ± 6.0 years old	participants enrolled in a physical activity intervention study	people with chronic obstructive pulmonary disease	Spearman	

Achamrah,et al..[14]	*2018*	*3,660 subjects (653 men and 3002 women)*	*BIA: Body Stat Quadscan 4000*<br>*DXA: Lunar Prodigy Advance (GE)*	*aged above 18 years old*	*patient*	*healthy but malnutrition obesity, eating disorder*	*Pearson's*	
Buch et al.[18]	2022	84 people (49 women)	BIA: InBody 770<br>DXA: GE Healthcare	71 ± 5 years old	patient	obese/overweight older adults with T2DM	ICC	
Vermeiren et al. [19]	2019	174 community dwellings (83 women, 91 men)	BIA: Bodystat® QuadScan 4000<br>DXA: Hologic 4500 QDR upgraded to Discovery	aged 80 and over	community	well-functioning persons	Pearson's	
Bosaeus et al. [20]	2013	117 subjects (72 males and 45 females)	BIA: ImpediMed DF50 single-frequency device<br>DXA: Lunar Prodigy	age 75 ± 4 years old, age range 70–93	patient	with a range of clinical conditions	Pearson's	
**NIBM-IBM**								

Kawai et al. [35]	2017	1239 (men: 511, women:728)	BIA: InBody 720 (InBody Inc., Seoul, Korea)<br>US: 6 MHz linear array transducer (Miru-Cube, Global Health, Kanagawa, Japan)	Mean 72.8 ± 5.3 years old	community-dwelling older adults	/	Pearson's	BIA-US
Hida et al. [36]	2018	201 subjects (male: 99 female: 102)	BIA: Inbody 720<br>US: Hitachi Aloka Medical	Mean 66.2 years old	community	healthy	Pearson's	
Ramírez-Fuentes et al. [37]	2019	35 men (with COPD: 18 healthy: 17)	BIA: Body Stat 1500<br>US: EsaoteMyLabfive	both groups 55–90 years old	community	COPD; healthy	Pearson's	
Wilkinson et al. [38]	2020	113 patients with Chronic Kidney Disorder (38% male)	BIA: InBody370;<br>InBody, Cerritos, CA<br>US: EUB-6500; Hitachi Medical Systems, Twinsburg, OH	Mean 62 years old	patient	Chronic Kidney Disorder	Pearson's	
Battaglia et al. [39]	2020	65 subjects (38 males)	BIA: AKERN EFG Plus<br>US: Philips Envisor C HD	HD: Mean 69 years old<br>Healthy: Mean 47.2 years old	patient	Chronic haemodialysis patients: healthy	Pearson's	
Simó-Servat et al. [40]	2023	32 subjects (75% female)	BIA: The BodyStat® 1500 MDD model<br>US:<br>Logiq P9 (GE Healthcare) equipment muscle-skeleton B-model	mean age: 49.15 ± 1.9 years old	patient	who underwent Bariatric Surgery	Pearson's	
Matsuzawa et al. [41]	2021	58 haemodialysis patients (22 females)	BIA: InBody 770<br>US: Xario 200, Toshiba Medical Systems, Japan	mean 77.5 years old	outpatient	58 haemodialysis patients	Pearson's	
Pietiläinen et al. [46]	*2012*	*19 people (12 females, 7 males)*	*BIA: Omron BF-500; Omron Medizintechnik*<br>*MRI: 1.5 T Philips MRI scanner*	*aged 20·2–48·6, mean age: 35·8*	*recruit via newspaper*	*obese adults*	*Pearson's*	BIA-MRI
Browning et al. [47]	*2012*	*120 participants (60 female and 60 male)*	*BIA:AB-140 (Tanita, Tokyo, Japan)*<br>*MRI:GE 1.5T HDx (GE Healthcare, Waukesha, WI)*<br>*sliceOmatic, version 4.3 software,*	*aged 18-79 years old*	*NA*	*lean-overweight-obese*	*Pearson's*	

			licensed by TomoVision (TomoVision, Montreal, Quebec, Canada)					
Wang et al. [48]	2013	200 subjects (100 male and 100 female)	BIA1: HBF 359 (Omron)<br>BIA2: BC 532 (Tanita)<br>MRI1: a General Electric (GE Healthcare, Milwaukee, WI, USA)<br>MRI2: a Siemens (Berlin and Munich, Germany ZedView 3.1 software (LEXI, Tokyo, Japan)	18–80 years old	patient	no chronic disease, implanted or ambulatory diagnostic or therapeutic device, and were not pregnant	intraclass correlation	
Chaudry et al. [49]	2020	63 male	BIA: BIA (InBody770; InBody, Seoul, South Korea)<br>MRI: a 3-T magnetic resonance system<br>(MAGNETOM Skyrafit;<br>Siemens Healthcare GmbH, Erlangen, Germany).	Group1: 21-36 years old (mean 28)<br>Group2: 70-86 years old (mean 76)	NA	NA	Pearson's	
Kiefer et al. [50]	2022	Among 335 included subjects (56.1% male)	BIA: (BIA 2000-S, Data-Input, Pöcking, Germany)<br>MRI: 3-Tesla Magnetom Skyra<br>(Siemens Healthineers, Erlangen, Germany)<br>Software MR LiverLab (Version VD13, Siemens Healthineers, Cary, USA)	56.3 ± 9.1 years old	NA	95 (28.4%) were obese (BMI ≥ 30 kg/m2).	Pearson's	
Gibson et al. [56]	2014	43 patients (62.8% male)	BIA: Bodystat 1500<br>CT: SliceOmatic software (v5 Tomovision	mean age 69.5 years old	patient	Weight-losing patients with CRC (colorectal cancer)	ICC	BIA-CT

Jo et al. [57]	2018	1,191 subjects (641 men and 521 women)	BIA: The InBody 770 (Biospace Co., Seoul, Korea)<br>CT: dual-source 128-slice CT scanner (Somatom Definition Flash, Siemens Healthcare, Forchheim, Germany),<br>a 64-slice CT scanner (Somatom Sensation 64, Siemens Healthcare),<br>a Discovery 710 PET-CT 128-slice scanner (General Electric Medical Systems, Milwaukee, WI, USA),<br>a Biograph TruePoint 40 PET-CT 40-slice scanner (Siemens Medical Solutions, Hoffman Estates, IL, USA),<br>a Discovery 600 PET-CT 16-slice scanner (General Electric Medical Systems, Milwaukee, WI, USA).	age >65 years old	patient	NA	not specified	
Ohara et al. [58]	*2020*	*110 chronic liver disease (CLD)*<br>*male: 71 (64.5%)*	*BIA: (InBody770; Inbody Japan Inc., Tokyo, Japan)*<br>*CT Brand not mentioned*<br>*sliceOmatic® software*	*range 21 to 90 years old*	*patient*	*chronic liver disease (CLD)*	*Spearman's*	
Grossberg et al. [59]	*2021*	*48 patients (40 men and 8 women)*	*BIA: FDA-cleared SECA mBCA 515 scale*<br>*CT: Brand not mentioned*	*aged ≥ 18 years old*	*patient*	*with head and neck cancer*	*Pearson's*	
Cao_et al._[60]	2022	606 included subjects (63.5% male)	BIA: Euromedix, Leuven, Belgium<br>CT: Revolution 256 and Lightspeed 64; GE Healthcare	59.7 ± 16.6 years old	patient	random	Pearson's	

Looijaard et al. [61]	2020	110 patients (68% male)	BIA: AKERN BIA 101 Anniversary<br>CT: Slice-O-matic versions 4.3 and 5.0	Mean age 59 ± 17 years old,	patient	mean APACHE II score 17 (11-25);	Pearson's	
Chen et al. [51]	2007	101 women (15 Hispanic-white, 1 African-American, 86 non-Hispanic-white women, and 2 women from ethnic backgrounds other than these listed)<br><br>postmenopausal female	MRI:3-T MRI scanner (model GE, General Electric<br>DXA: The Hologic QDR 4500w	aged 50–79 years old	N/A	The exclusion criteria were: 1) weighed >113.6 kg (250 pounds, due to weight restrictions by the DXA machine); 2) inability to undergo an MRI scan due to metal implants, extreme claustrophobia, or recent surgery; 3) unable to lie supine for 30 min; and 4) unable to raise her arms over her head for 15 min.	Pearson's	DXA-MRI
Maden-Wilkinson et al. [27]	*2013*	*total 91 subjects: younger: 20 males 18 females<br>older: 25 males 28 females*	*MRI:G-Scan, Esaote, Genova, Italy<br>DXA: Lunar Prodigy Advance GE*	*young: 20 men; 22.4±3.1 years old; 18 women; 22.1±2.0 years old<br>old: 25 men; 72.3±4.9 years old; 28 women; 72.0±4.5 years old*	*community*	*healthy*	*Pearson's*	
Yang et al. [52]	2016	190 subjects (58 males and 132 females)	MRI: Siemens Magnetom Trio, Germany<br>DXA: (Discovery APEX 13.3; Hologic, Bedford, MA, USA	aged >50 years old	community	independent in their activities of daily living with no functional issues	Pearson's	
Tavoian et al. [53]	*2019*	*10 males and 16 females*	*MRI: Esaote G-Scan Brio, Genoa, Italy*	*29.2 ± 9.5 years old*	*N/A*	*N/A*	*Pearson's*	

			DXA: Hologic Discovery QDR model Series, Waltham, MA, USA					
Brown et al. [54]	2022	36 people with Type 2 diabetes (T2D)(17 men, 19 women)	MRI: Siemens Medical Solutions, Erlargen, Germany<br>DXA: Lunar DXA, GE Healthcare	age 48.4 ± 8.2 years old	N/A	be ambulatory but non-exercising and non-smoking	Pearson's	
Cho et al. [55]	2022	68 patients: females (83.3%)	MRI: GE Healthcare<br>DXA: N/A	mean age was 77.9 ± 7.0 years old	patient	patients with probable Alzheimer's disease (AD) without weakness were included. T	Pearson's	
Bredella et al. [62]	*2010*	*91 premenopausal women (34 obese, 39 with AN, and 18 lean controls).*	*CT: software Alice, version 4.3.9; Parexel, Waltham, MA and Accuview, version 3.130; AccuImage Diagnostics, San Francisco, CA<br>DXA: Hologic, Waltham, MA*	*18–45 years old*	*N/A*	*Overweight or obese subjects had a BMI ≥25 kg/m2, and lean controls had a BMI ≥19 kg/m2 and <25 kg/m2*	*N/A*	DXA-CT
Kim et al. [63]	2023	77 males and 43 females	DXA: Discovery W DEXA and APEX software ver. 13.6.1.1; Hologic, Inc.<br>CT: Somatom Definition Edge and Somatom Definition FLASH [Siemens Healthineers]; Brilliance 64 [GE Healthcare]	aged 61.4 ± 11.0 years old	patient	who underwent gastrectomy	Pearson's	
Yoo et al. [64]	*2022*	*a total of 100 adults (60 women)*	*DXA: Lunar Prodigy Advance; GE Healthcare<br>CT: IQon Spectral CT; Philips Healthcare*	*aged 20–69 years old (mean age: 44.9)*	*recruited*	*healthy*	*Pearson's*	
Tsukasaki et al.[65]	2020	A total of 1,818 subjects (943 men and 875 women)	DXA: (Hologic, Bedford, MA, USA)<br>CT: (X-Vision; Toshiba, Tokyo, Japan, and SOMATOM Sensation 64; Siemens, Munich, Germany)	age 40–89 years old	community	random	Pearson's	
Berger et al. [43]	*2015*	*54 adults (27 women) and 51 older adults (26 women)*	*US: General Electric Logiq ultrasonographer<br>DXA: Lunar General Electric iDEXA equipment.*	*54 adults of both genders, aged 20–55 years old and 51 adults older than 60 years old.*	*community*	*healthy*	*Pearson's*	DXA-US

Zhu et al. [44]	2019	265 elder community dwellers from China (97 males and 168 females)	US: A B-mode ultrasound (Philips iU Elite, Bothell, WA, USA) DXA: DXA scanner (Hologic Inc., Bedford, MA, USA)	60 years old or older	community	who participated in the annual health screening	Pearson's	
Álvarez et al. [45]	2021	57 participants older than 70 years old (33 female)	US: General Electric Logic F6 device DXA: GE Healthcare Lunar Prodigy Advance DXA Scan	older than 70 years old Median age was 78.9 years old	N/A	walk independently	ICC	
Souza et al. [34]	2018	100 patients (59 females)	US: Siemens Sonoline G40 (Korea, 2007) DXA: GE Lunar Prodigy Primo equipment	aged 65 years old or older	patients	nondialysis chronic kidney disease were evaluated	Pearson's	
**IBM-IBM**								
Khan et al. [21]	2019	10 patients with renal cell carcinoma (RCC) and CT abdomen/pelvis. An additional sample of 9 patients with RCC and both CT and T2-weighted (T2w) MRI abdomen/pelvis, total 19 subjects (15 males, 4 females)	The MRI studies were all done using Siemens 1.5 Tesla scanners. SMA was segmented using Slice-O-Matic®.	N/A	patients	with RCC	ICC	MRI-CT
Zwart et al. [22]	2020	125 patients (72% male)	MRI: MRI scanners (1.5-T Area or 3-T Prisma or Skyra). CT: Siemens Healthcare CT (Biograph64, SOMATOM Force, SOMATOM Open, SOMATOM Definition AS or SOMATOM Definition Flash)	mean age of 63 (42–82) years old	patients	head and neck cancer	Pearson's	

Faron et al. [23]	2020	50 patients (19 females, 31 males)	CT-scanner (Brilliance iCT SP 128 CT, Philips Healthcare, Best, the Netherlands)<br>MRI: 1.5 T scanner (Ingenia 1.5 T, Philips Healthcare, Best, the Netherlands)	61 ± 6 years old	patients	lung cancer screening program	Pearson's	
Lee et al. [24]	2021	106 patients (22 women and 84 men)	CT: head and neck CT scans on 64- or 256-channel scanners (Brilliance, the IQon, and the iCT, Philips Healthcare, Best, The Netherlands).<br>MRI: 3-T MR scanner (Achieva, Ingenia, and Ingenia CX; Philips Medical Systems, Best, The Netherlands)	mean age, 66.4 years old	patients	with newly diagnosed HNSCC were included.	ICC and the Pearson	
Dupont et al. [25]	*2001 (only abstract)*	*6 healthy human subjects (3 men and 3 women)*	*CT: High-Speed Advantage system (both from GE Medical Systems, Milwau-kee, WI)*	*24–51 years old*	*N/A*	*healthy*	*N/A*	
Wang et al. [26]	2021	32 patients (15 males and 17 females)	MRI: a 3.0 Tesla MRI system (Discovery MR 750, GE Signa advantage HDxt, GE Healthcare, USA<br>CT: a Philips 256 iCT scanner (Philips Healthcare, Amsterdam, Netherlands)	mean age was 57.2 years old	patients	diagnosed with various kidney diseases	ICC and the Pearson	
Abe et al. [28]	*2017*	*10 young and middle-aged adults 8 men and 2 women*	*US: B-mode ultrasound (Aloka SSD-500, Tokyo, Japan)*<br>*MRI: Hitachi APERTO Lucent (0·4-Tesla open permanent magnet, Hitachi Medical Corporation, Tokyo, Japan)*	*mean age 31 (SD 14) years old*	*volunteers*	*Subjects had no orthopaedic abnormalities (e.g., surgery or trauma) in their upper extremities.*	*Pearson's*	MRI-US
Mul et al.[29]	*2018*	*27 patients (17 males, 10 females)*	*MRI: 3-Tesla MR system (TIM Trio; Siemens, Erlangen, Germany).*<br>*US: Esaote MyLabTwice ultrasound scanner (Esaote SpA, Genoa, Italy) 8–14 MHz broadband linear transducer with a 53-mm footprint*	*18 years old and older*	*patients*	*FSHD patients*	*Pearson's*	
Franchi et al. [30]	*2018*	*9 males*	*US: Mylab 25; Esaote Biomedica, Genova, Italy*	*age = 24 ± 2 years old*	*volunteers*	*active, young healthy*	*Pearson's*	

			MRI: MRI (GE, 3T 750 Discovery, Chalfont Saint Giles UK)					
Giles et al. [31]	*2014*	*5 participants (2 men, 3 women) 10 limbs*	*US: HDI3000 by Advanced Technology Laboratories, California*<br>*MRI: (3.0T Philips (Ingenia, Philips Medical Systems, The Netherlands*	*aged 24–37 years old*	*patients*	*with unilateral patellofemoral pain for greater than 6 weeks*	*Pearson's*<br>*Spearman's*	
Seymour et al [32]	2009	56 subjects (48.2% male)(Main study: 30 patients with stable COPD (male: female 16:14) and 26 healthy volunteers participated in the study (male: female 11:15), (18 participants agreed to an additional CT scan of the quadriceps.))	US: B-mode ultrasonography using an 8 MHz 5.6 cm linear transducer array (PLM805, Toshiba Medical Systems, Crawley, UK)<br>RFCSA was calculated via a planimetric technique (Nemio, Toshiba Medical Systems)<br>CT: Siemens SOMATOM Sensation 64-slice scanner, Siemens SIENET software (MagicView VE 40)	Mean (SD) age 63 for 26 healthy volunteers and Mean (SD) 67 for 30 patients	patients; community	COPD; healthy	ICC	CT-US
Thomaes et al. [33]	2012	45 CAD patients (44 males, 1 female)	US: Siemens Vivid 07 GE)<br>with a 12 MHz linear array transducer (12 L transducer GE).<br>CT: Siemens Sensation 16	age: 68.4 ± 6.2 years old	patients	participating in sporting activities of a maintenance programme for patients with cardiovascular disease,	Pearson's, ICC	
Souza et al. [34]	2018	100 patients (59 females)	US: Siemens Sonoline G40 (Korea, 2007)<br>CT: Siemens Emotion device (Germany, 2007)	73.5±9.22 years old	patients	With nondialysis chronic kidney disease	Pearson's	

SUPPLEMENTARY DATA

Table 6. Abbreviations of terminology for whole body/muscular/fat indexes

Abbreviation	Full Terminology
ADI	Adipose Index
AFM	Arm Fat Mass
ALM	Appendicular Lean Mass
ASM(I)	Appendicular Skeletal Mass (Index)
ASMM/ASM	Appendicular Skeletal Muscle Mass
AMMIFat-free	Fat-free Abdominal Skeletal Muscle Mass Index
AMMITotal	Total Abdominal Skeletal Muscle Mass Index
AMMI	Abdominal Skeletal Muscle Mass Index
BCM	Body Cell Mass
BMI	Body Mass Index
BFP	Body Fat Percentage
CKD	Chronic Kidney Disease
CSA	Cross-Sectional Area
CVD	Cardiovascular Disease
BFM	(Body) Fat Mass
FM	Fat Mass
FFMI	Fat-Free Mass Index
FMI	Fat Mass Index
FMMP	Fat-Free Mass Percentage
FFM	Fat-Free Mass
IMF	Intermuscular Fat
LBM	Lean Body Mass
LFM	Leg Fat Mass
LM	Lean Mass
LSMA	Lumbar Skeletal Mass Area
LSTM	Lean Soft Tissue Mass
MM	Muscle Mass
MT	Muscle Thickness
MV or VOL	Muscle Volume
PBF	Percentage Body Fat
PF	Percentage Fat
PMI	Psoas Muscle Mass Index
QRFM	Quadriceps Rectus Femoris
QMVC	Quadriceps Strength was assessed via Isometric Maximum Voluntary Contraction
RF	Rectus Femoris
SM	Skeletal Muscle
SMA	Skeletal Muscle Area
SMI	Skeletal Muscle Mass Index
SMP	Skeletal Muscle Percentage
SMM	Skeletal Muscle Mass
SFT	Subcutaneous Fat Tissue
SF	Subcutaneous Fat
TAAT	Total Abdominal Adipose Tissue
TBF	Total Body Fat
TF(M)	Trunk Fat (Mass)
TMM	Total Muscle Mass
(T)MT	(Temporalis) Muscle Thickness; Thigh Muscle Thickness
TPF	Total Percentage Fat
TwQ	Twitch Tension
VAT	Visceral Adipose Tissue
(V)FA	(Visceral) Fat Area
VTAT	Volume of Visceral Adipose Tissue
AVAT	Area of Visceral Adipose Tissue
VF	Visceral Fat
VFL	Visceral Fat Level
VI	Vastus Intermedius
VL	Vastus Lateralis

© 2025. Zhang D et al. Published online at http://www.aginganddisease.org/EN/10.14336/AD.2025.0145

VM	Vastus Medialis

Table 7. Abbreviation for Subcategories of Global/Partial and Muscle/Fat

Abbreviation	Definition	Index Example
G	Global	(total) FFM, ALM, Dry LM, BCM, FFMP, Lean Body Mass Index, Whole body LSTM, BMI
P	Partial	Arms Lean Body Mass,<br>Legs Lean Body Mass,<br>Trunk Lean Body Mass,<br>ALM of gastrocnemius medialis,<br>LBM in the upper limbs,<br>LBM in the lower limbs,<br>Thigh LM
PM	Partial-Muscle	Abdominal SMA,<br>MT of Temporalis/ supraspinatus/deltoid muscle/ulna/radius/ quadriceps/VL/(right/left) RF/thigh/Top-quadriceps rectus femoris (QRFM)/ Mid-QRFM/ Low-QRFM/ gastrocnemius medialis/ temporalis,<br>CSA of forearm/RF,<br>Mean z-score of<br>total legs/RF,<br>Muscle Volume (VOL) of VL, Mid-thigh,<br>Diameter of RF,<br>Leg muscle mass,<br>Single-slice thigh<br>estimates of whole-body SMM,<br>LSMA
PF	Partial-Fat	Arms Fat Mass,<br>Legs Fat Mass,<br>Trunk Fat Mass,<br>Mean fat fraction% of RF,<br>Mean fat fraction% of total leg,<br>Abdominal subcutaneous<br>fat tissue thickness,<br>SF,<br>Visceral Adipose Tissue Area, Fat fraction of total abdominal volume of interest, Fat fraction of visceral volume of interest, SF of thigh, (Intermuscular Fat)<br>IMF of thigh
GM	Global-Muscle	ASMI, SMM, SMI, TMM, ASM, Skeletal Muscle Percentage (SMP), (Abdominal skeletal muscle mass index)<br>AMMI, Whole body SMM, SM mass, muscle mass
GF	Global-Fat	BFP, FM, TPF, PF, PBF, Visceral Fat, Visceral Index, Total abdominal adipose tissue, Visceral Fat Level, VFL, Total abdominal<br>adipose tissue, Body Fat Mass (BFM), Volume of total adipose tissue, Visceral Fat Area

Table 8. Modern Modalities for Sarcopenia Assessment Recommendation Table

Approach	Patient's Characteristics	Available Resources	Specific Assessment Goals
BIA	Suitable for daily monitoring and management of various body composition indicators to obtain quick and instant results for (daily) routine examination. It helps to monitor changes during sarcopenia treatment, including diet and exercise interventions.	Due to its accessibility and affordability (price range from low to high), can be approached in various settings such as hospitals, community clinics, gyms, and	Primarily used to measure the overall body condition, particularly indicators related to BFP and BMI, but it can also assess metrics related to muscle or fat, such as FFM, ASM, and SMI.

© 2025. Zhang D et al. Published online at http://www.aginganddisease.org/EN/10.14336/AD.2025.0145

		even at home for daily routine measurements.	
DXA	Suitable for assessing body composition while simultaneously measuring bone density. However, it is not ideal for routine daily testing due to the associated low radiation exposure risk.	DXA could be visited by specialized DXA testing centres. Some general health check-up centres also offer DXA testing services.	Examine whole-body parameters when needed for higher accuracy and for bone-related parameters such as bone density conditions.
MRI	Typically used for patients requiring diagnostic evaluation of a specific disease that needs MRI tests while simultaneously assessing partial muscle or fat. Due to the high cost, MRI is not suitable for routine tracking of general body composition or muscle/fat changes.	MRI could be visited in specialized MRI testing centres and hospitals.	Able to access partial muscle and fat condition in the abdominal area and limbs such as MT and CSA of a particular muscle.
CT	Typically used for patients requiring diagnostic evaluation of a specific disease that needs CT tests while simultaneously assessing partial muscle or fat. Due to the radiation risk, CT is unsuitable for routine tracking of general body composition or muscle/fat changes.	CT could be visited in specialized CT testing centres and hospitals	Able to access partial muscle and fat condition in the abdominal area and limbs such as diameters and CSA of a particular muscle.
US	Available for patients who need routine monitoring of muscle and fat conditions and effectively track improvements in specific muscle conditions over time due to its non-invasive nature, free from worries of radiation, and relatively low cost.	US could be visited in specific US testing centres and hospitals. Some palm-sized US devices are also approachable for community settings.	Able to access instant results of partial muscle and fat conditions in the abdominal area and limbs, such as MT and CSA of a particular muscle.

© 2025. Zhang D et al. Published online at http://www.aginganddisease.org/EN/10.14336/AD.2025.0145

SUPPLEMENTARY DATA

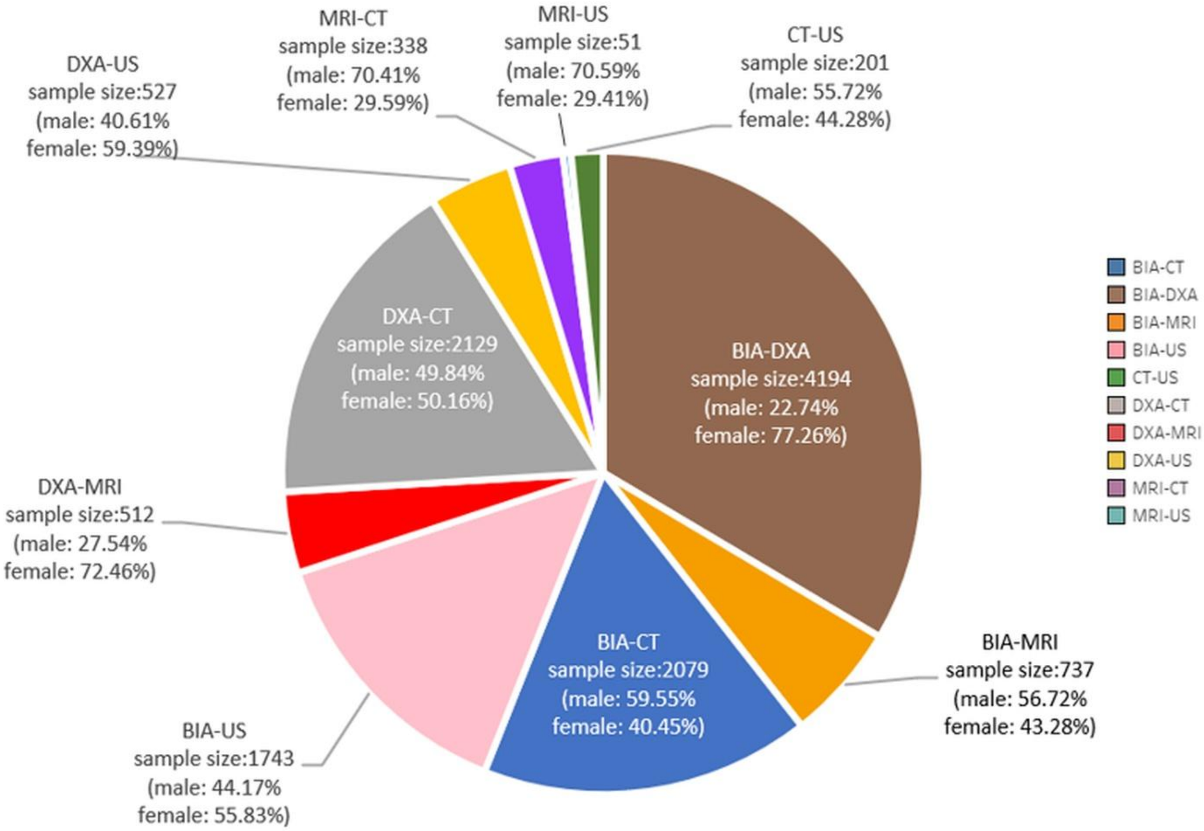

Figure 1. A pie chart providing information of the sample size and gender distribution among the ten pairs of technologies (BIA-DXA, BIA-MRI, BIA-CT, BIA-US, DXA-MRI, DXA-CT, DXA-US, MRI-CT, MRI-US, CT-US)

© 2025. Zhang D et al. Published online at http://www.aginganddisease.org/EN/10.14336/AD.2025.0145

SUPPLEMENTARY DATA

Table 9. Correlations of Muscle and Sarcopenia-related Factors

Category	Parameter 1	Parameter 2	correlation	Author
BIA-DXA (*Tengvall, Kyle, and Janssen are three authors who generated the equation for calculating skeletal muscle mass) Average: 0.931	ASMI (GM: Global, Muscle)	ASMI (GM: Global, Muscle)	0.965	Buch et al. [18]
	SMM (Tengvall et al.*) (GM: Global, Muscle)	SMM (GM: Global, Muscle)	0.915	
	SMM (Kyle et al.*) (GM: Global, Muscle)	SMM (GM: Global, Muscle)	0.939	
	SMM (Janssen et al.*) (GM: Global, Muscle)	SMM (GM: Global, Muscle)	0.906	Bosaeus et al. [20]
MRI-CT Average: 0.966	Abdominal SMA (PM: Partial, Muscle)	Abdominal SMA (PM: Partial, Muscle)	0.997	Khan et al. [21]
	Total CSA of head-and-neck muscles at the C3 level (PM: Partial, Muscle)	Total CSA of head-and-neck muscles at the C3 level (PM: Partial, Muscle)	0.987	
	SMI (GM: Global, Muscle)	SMI (GM: Global, Muscle)	0.997	Zwart et al. [22]
	Paraspinal SMA (PM: Partial, Muscle)	Paraspinal SMA (PM: Partial, Muscle)	0.930	Faron et al. [23]
	MT of Temporalis (PM: Partial, Muscle)	MT of Temporalis (PM: Partial, Muscle)	0.894	Lee et al. [24]
	MT of supraspinatus (PM: Partial, Muscle)	MT of supraspinatus (PM: Partial, Muscle	0.960	
	MT of deltoid muscle (PM: Partial, Muscle)	MT of deltoid (PM: Partial, Muscle)	0.970	Dupont et al. [25]
	Abdominal SMA (PM: Partial, Muscle)	Abdominal SMA (PM: Partial, Muscle)	0.995	Wang et al. [26]
MRI-US Average: 0.874	CSA of forearm	MT of ulna	0·937–0·946	Abe et al. [28]
	CSA of forearm	MT of radius	0·884–0·891	
	CSA of vastus lateralis (VL) (PM: Partial, Muscle)	MT of VL (PM: Partial, Muscle)	0.820	
	Muscle Volume (VOL) of VL (PM: Partial, Muscle)	MT of VL (PM: Partial, Muscle)	0.760	Franchi et al.[30]
	MT of RF (PM: Partial, Muscle)	MT of RF (PM: Partial, Muscle)	0.858	
	CSA of RF (PM: Partial, Muscle)	MT of RF (PM: Partial, Muscle)	0.897	
	MT of quadriceps (PM: Partial, Muscle)	MT of quadriceps (PM: Partial, Muscle)	0.915	Giles et al. [31]
CT-US Average: 0.875	CSA of RF (PM: Partial, Muscle)	CSA of RF (PM: Partial, Muscle)	0.880	Seymour et al.[32]
	Diameter of RF (PM: Partial, Muscle)	Diameter of RF (PM: Partial, Muscle)	0.920	Thomaes et al. [33]
	CSA of RF (PM: Partial, Muscle)	CSA of RF (PM: Partial, Muscle)	0.826	Souza et al [34]

© 2025. Zhang D et al. Published online at http://www.aginganddisease.org/EN/10.14336/AD.2025.0145

	SMI (Before control for age) (GM: Global, Muscle)	MT (Before control for age) (PM: Partial, Muscle)	Men: 0.454 Women: 0.414	
	SMI (After control for age) (GM: Global, Muscle)	MT (After control for age) (PM: Partial, Muscle)	Men: 0.445 Women: 0.395	Kawai et al. [35]
	ASMI (GM: Global, Muscle)	MT of thigh (PM: Partial, Muscle)	0.380	Hida et al. [36]
	ASM (GM: Global, Muscle)		0.650	
	TMM (GM: Global, Muscle)	CSA of RF (PM: Partial, Muscle)	0.660	Wilkinson et al. [38]
	SMI (GM: Global, Muscle)	TMT (PM: Partial, Muscle)	pre-surgical 0.350	
	SMI (GM: Global, Muscle)	TMT (PM: Partial, Muscle)	post-surgical 0.380	Simó-Servat et al. [40]
	Leg muscle mass (PM: Partial, Muscle)		0.685	
	ASM (GM: Global, Muscle)		0.693	
BIA-US Average: 0.517	ASM (GM: Global, Muscle)	CSA of RF (PM: Partial, Muscle)	0.698	Matsuzawa et al.[41]
	MT of gastrocnemius medialis (PM: Partial, Muscle)	ALM of gastrocnemius medialis (P: Partial)	Longitudinal plane: 0.689 Transverse plane: 0.546	Álvarezet al. [45]
	CSA of RF (using US and CT)	LBM in the upper limbs (P: Partial)	0.286	
DXA-US Average: 0.448	(PM: Partial, Muscle)	LBM in the lower limbs (P: Partial)	0.271	Souza et al. [34]
	Skeletal Muscle Percentage (SMP) (HBF) (GM: Global, Muscle)	SMP (GM: Global, Muscle)	0.850	Wang et al. [48]
BIA-MRI Average: 0.716	SMI (GM: Global, Muscle)	SMI (GM: Global, Muscle)	0.581	Kiefer et al. [50]
	Whole-body SMM (GM: Global, Muscle)	Whole-body SMM (GM: Global, Muscle)	Male: 0.940 Female: 0.940	
	Whole-body SMM (GM: Global, Muscle)	Single-slice thigh estimates of whole-body SMM (PM: Partial, Muscle)	Male: 0.880 Female: 0.870	Brown et al. [54]
DXA-MRI Average:0.8018	ASM (GM: Global, Muscle)	MT of temporalis (PM: Partial, Muscle)	0.379	Cho et al. [55]
	ASM (GM: Global, Muscle)	LSMA (PM: Partial, Muscle)	Male: 0.724 Female: 0.645	
	ASM (GM: Global, Muscle)	LSMA (PM: Partial, Muscle)	0.898	
	ASM (BMI adjusted) (GM: Global, Muscle)	LSMA (BMI adjusted) (PM: Partial, Muscle)	0.858	Jo et al.[57]
	SMI (GM: Global, Muscle)	SMI (GM: Global, Muscle)	0.610	
	SMI (>6 months post-intervention for CLD) (GM: Global, Muscle)	SMI (>6 months post-intervention for CLD) (GM: Global, Muscle)	0.510	Ohara et al. [58]
BIA-CT Average: 0.777	SM mass (GM: Global, Muscle)	SM CSA (lumbar region) (PM: Partial, Muscle)	Head&neck cancer: 0.969	Grossberg et al. [59]

© 2025. Zhang D et al. Published online at http://www.aginganddisease.org/EN/10.14336/AD.2025.0145

	SMI<br>(GM: Global, Muscle)	SMI<br>(lumbar region)<br>(PM: Partial, Muscle)	Head&neck cancer:<br>0.948	
	MM<br>(*Talluri)<br>(GM: Global, Muscle)	MM at L3 level<br>(PM: Partial, Muscle)	0.834	Looijaard et al. [61]
DXA-CT<br>Average:0.737	ASMI<br>(GM: Global, Muscle)	ASMI<br>(GM: Global, Muscle)	0.794	Kim et al. [63]
	SMI<br>(GM: Global, Muscle	SMI<br>(GM: Global, Muscle	Men: 0.800<br>Women: 0.710	Tsukasak et al.[65]
		CSA of quadriceps<br>(PM: Partial, Muscle)	Men: 0.750<br>Women: 0.630	

© 2025. Zhang D et al. Published online at http://www.aginganddisease.org/EN/10.14336/AD.2025.0145
